# High‐Performance Pure Water‐Fed Anion Exchange Membrane Water Electrolysis with Patterned Membrane via Mechanical Stress and Hydration‐Mediated Patterning Technique

**DOI:** 10.1002/advs.202409563

**Published:** 2024-12-16

**Authors:** Yeonjae Lee, Sungjun Kim, Yoseph Shin, Yeram Shin, Seongmin Shin, Sanghyeok Lee, Minseop So, Tae‐Ho Kim, Sehkyu Park, Jang Yong Lee, Segeun Jang

**Affiliations:** ^1^ School of Mechanical Engineering Kookmin University Seoul 02707 Republic of Korea; ^2^ Hydrogen Energy Research Center Korea Research Institute of Chemical Technology (KRICT) Daejeon 34114 Republic of Korea; ^3^ Department of Chemical Engineering Kwangwoon University Seoul 01897 Republic of Korea; ^4^ Department of Chemical Engineering Konkuk University Seoul 05029 Republic of Korea

**Keywords:** anion exchange membrane water electrolysis, dehydration, enlarged interfacial area, patterned membrane, pure water fed

## Abstract

Despite rapid advancements in anion exchange membrane water electrolysis (AEMWE) technology, achieving pure water‐fed AEMWE remains critical for system simplification and cost reduction. Under pure water‐fed conditions, electrochemical reactions occur solely at active sites connected to ionic networks. This study introduces an eco‐friendly patterning technique leveraging membrane swelling properties by applying mechanical stress during dehydration under fixed constraints. The method increases active sites by creating additional hydroxide ion pathways at the membrane‐electrode interface, eliminating the need for additional ionomers in the electrode. This innovation facilitates ion conduction via locally shortened pathways. Membrane electrode assemblies (MEAs) with patterned commercial membranes demonstrated significantly improved performance and durability compared to MEAs with conventional catalyst‐coated substrates and flat membranes under pure water‐fed conditions. The universal applicability of this technique was confirmed using in‐house fabricated anion exchange membranes, achieving exceptional current densities of 13.7 A cm^−2^ at 2.0 V in 1.0 M potassium hydroxide (KOH) and 2.8 A cm^−2^ at 2.0 V in pure water at 60 °C. Furthermore, the scalability of the technique was demonstrated through successful fabrication and operation of large‐area cells. These findings highlight the potential of this patterning method to advance AEMWE technology, enabling practical applications under pure water‐fed conditions.

## Graphical abstract

An eco‐friendly large‐area patterning technique is developed that employs membrane swelling properties. With the advantages of facilitated ion conduction and secured more active sites, excellent performance of AEMWE with 13.7 A cm^−2^ (1 m KOH) and 2.8 A cm^−2^ (pure water) at 2.0 V is achieved. The practical applicability of the method is validated with a large‐area single‐cell (68.75 cm^2^)

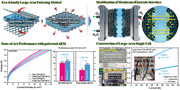

## 1 Introduction

The transition to sustainable energy systems necessitates the development of efficient and cost‐effective hydrogen production methods for. As a clean energy carrier, hydrogen is crucial for decarbonization of various industrial processes.^[^ ^1^ ^]^ Anion exchange membrane (AEM) water electrolysis (AEMWE) is an emerging technology that combines the advantages of both alkaline and proton exchange membrane water electrolysis technologies. It utilizes a solid anion‐conducting polymer membrane to create a locally alkaline environment, enabling earth‐abundant catalysts use, low gas crossover rates, and high‐pressure hydrogen production.^[^ ^2^ ^]^ Despite advancements in hydroxide conductivity and alkaline stability, AEMWEs face challenges in industrial applications because of their low water electrolysis performance, limited long‐term durability, and the necessity for a supporting electrolyte incompatible with pure water‐fed conditions.^[^ ^3^ ^]^ Using pure water instead of a supporting electrolyte offer several advantages, including enhanced system safety, simplified systems, plant‐level engineering, and the elimination of efficiency losses caused by shunt currents.^[^ ^4^ ^]^ However, the performance and durability of AEMWEs are significantly reduced without a supporting electrolyte, potentially making them less competitive than proton exchange membrane water electrolysis (PEMWE).^[^ ^5^ ^]^ This reduction is due to decreased local pH at the catalyst/ionomer interface,^[^ ^3c^ ^]^ leading to reduced catalyst activity,^[^ ^6^ ^]^ morphological and structural changes in the catalyst,^[^ ^7^ ^]^ and accelerated ionomer oxidation, which causes poisoning issues.^[^ ^8^ ^]^ In water‐fed AEMWE, electrochemical reactions occur exclusively at specific locations known as “triple‐phase boundaries” (TPBs), which are characterized by the presence of electron conductors, active catalysts, anionic carriers, and pathways for reactants and products. The number of TPBs secured directly affects high performance, requiring advanced membrane electrode assembly (MEA) design and fabrication methods.^[^ ^5^, ^9^ ^]^

MEA fabrication for AEMWE commonly involves three methods: catalyst‐coated substrate (CCS), catalyst‐coated membrane (CCM), and direct membrane deposition (DMD).^[^ ^4b^ ^]^ The CCS procedure, the most prevalent, necessitates depositing catalyst slurry on a conductive porous transport layer. This produces a large number of MEAs in the industry. However, CCS typically suffers from low catalyst usage efficiency and weak electrode‐AEM bonding.^[^ ^10^ ^]^ This can lead to delamination at the interface and AEMWE performance degradation, especially in low‐concentration supporting electrolyte or pure water as the feed.^[^ ^11^ ^]^ In contrast, CCM allows for more effective utilization of the catalyst and improved adhesion between the electrode and AEM by applying the catalyst ink directly to the AEM surface.^[^ ^12^ ^]^ This method requires less catalyst loading and demonstrates superior electrochemical performance compared to the CCS. Novel approaches have been developed to enhance the performance of CCM‐type MEA by structurally modifying the membrane surface to increase the electrode–AEM interfacial area.^[^ ^13^ ^]^ Modified AEM with surface patterns offer significant advantages over conventional flat‐sheet AEMs in terms of ion transport, water management, and catalyst utilization.^[^ ^12^, ^14^ ^]^ Maximizing the interfacial contact area between the membrane and the electrode through a patterned AEM is beneficial for increasing the number of OH‐ transport pathways from the cathode to the anode in pure water‐fed AEMWE operations. OH^−^ transport relies solely on the ionomer network in the electrode and AEM because the hydroxide is primarily located around the ionomer's cationic group. However, most commercially available AEMs, such as FAA‐3 and PiperION, are made of aromatic hydrocarbon polymers with a glass transition temperature exceeding 200 °C.^[^ ^4^, ^15^ ^]^ This high temperature complicates the use of conventional thermal imprinting methods for creating patterns on polymeric membranes. Lee et al.,^[^ ^12b^ ^]^ proposed an alternative method involving pouring and drying lab‐synthesized ionomer (i.e., poly‐(dibenzyl‐co‐terphenyl piperidinium)) solution onto a patterned silicon wafer, achieving remarkable performance of 2 A cm^−2^ at 2.0 V under pure water condition. However, this approach is unsuitable for commercial or large‐area AEM films due to challenges in achieving high uniformity during the drying process, which hampers scalability of the film area. The DMD method facilitates effective interaction between the catalyst and AEM but is limited to small‐scale preparation.^[^ ^16^ ^]^ It involves depositing or growing the catalyst onto a substrate without using an ionomer, followed by a thin AEM layer onto the CCS method. An in situ‐produced AEM layer allows for efficient utilization of a nanostructured catalyst and enhances the interfacial contact area between the electrode and the AEM. Wang et al.^[^ ^16a^ ^]^ recently introduced a new 3D ordered MEA composed of highly porous catalyst layers of NiCo@FeNi LDH anode layer with vertical channels and a lab‐synthesized deposited membrane (i.e., poly(alkyl‐terphenyl piperidinium), achieving current densities of 3.1 A cm^−2^ at 2.0 V under pure water fed condition. Therefore, to enhance AEMWE efficiency with pure water feeding, expanding the three‐phase interface by modifying the structure of the membrane‐electrode interface is crucial. This necessitates the development of a new patterning technology that utilizes readily available materials and facilitate large areas production.

This study proposes an effective method for fabricating dual‐sided patterned AEMs to improve AEMWE devices efficiency, particularly under pure water‐fed conditions. Unlike traditional methods such as 3D printing,^[^ ^17^ ^]^ laser ablation,^[^ ^18^ ^]^ photolithography,^[^ ^19^ ^]^ sacrificial templating,^[^ ^13c^ ^]^ thermal imprinting,^[^ ^13b^ ^]^ and casting and drying of prepared molds.^[^ ^12b^ ^]^ which are not suitable for modifying hydrocarbon‐based AEMs and large‐area applications—this study employs high water uptake and dimensional changes of AEMs to soften the mechanically rigid membranes and create space for structural deformation. Large‐area roll‐type thin metal meshes are utilized to sandwich commercial AEMs, applying mechanical stress in a fixed‐constraint environment to induce mechanical creep deformation of the AEM. To prevent immediate shape recovery after removing the mechanical load, a dehydration procedure was conducted on the assembly, thereby restoring the original high mechanical characteristics of the AEM. The effect of varying hydration conditions on the AEM mechanical characteristics of was extensively studied to improve pattern fidelity. The AEMWE cell with patterned membranes showed a substantial enhancement in performance and durability when compared to cells with traditional flat membranes under pure water‐fed condition, regardless of the AEM used (commercial and in‐house fabricated AEMs). The notable enhancement in the performance of the patterned membrane underscores the effectiveness of the interlocking mechanism between the membrane‐electrode by patterning, which facilitates ion transport and minimizes polarization loss in a cell. Furthermore, the scalability of the patterning process was demonstrated by successfully fabricating large‐area patterned membrane and operating large‐size cells with an active area of 68.75 cm^2^.

## 2 Results and Discussion

### 2.1 Dual‐Side Patterned Membrane using Mechanical Stress and Dehydration‐Mediated Patterning Technique

**Figure 1:**
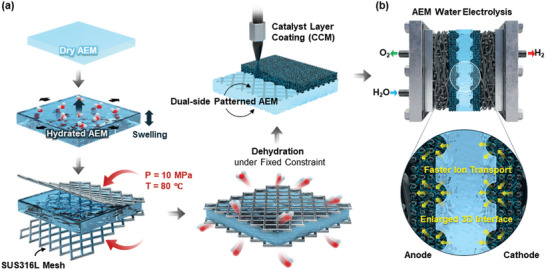
Schematics of a) fabrication process of dual‐side patterned AEM, and MEA with patterned AEM via direct catalyst deposition method, and b) Effects of dual‐side patterned AEM for AEMWE.

Commercially available AEMs, such as PiperION, have been extensively employed in AEMWE research because of their superior electrochemical properties. However, there have been limited research on the patterning these commercial AEMs because modifying the structure of membranes fabricated with aromatic hydrocarbon polymers, which have high glass transition temperatures and mechanical rigidity, has been challenging.^[^ ^4^, ^15^ ^]^ This study introduces a novel, environmentally friendly technique involving the mechanical deformation of a swollen, hydrated AEM material, which is plasticized with water. The technique applies fixed constraints and mechanical stress during the dehydration process. The fabrication process for the dual‐sided microstructured AEM is illustrated in **Figure** **1**a. Initially, a plasticized, hydrated, swollen‐AEM (i.e., PiperION membrane) was prepared at 80 °C for 24 h and positioned between two molds of SUS‐316L meshes. A mechanical pressure of 10 MPa is then applied at 80 °C to the sandwich‐like assembly. Under clamped constraint with two vertically oriented meshes, the structural creep deformation of the plasticized AEM occurs along its thickness direction as the meshes are inserted into the surface of both ends of the AEM.^[^ ^20^ ^]^ Following this, the sandwich assembly underwent a dehydration process for 2 h while maintaining mechanical pressure and was subjected to further deformation. During this process, permanent structural creep deformation was induced and prevented substantial immediate creep recovery upon mold removal by enhancing the AEM's inherent strong mechanical rigidity through complete dehydration (i.e., by reducing the flexibility of the AEM).^[^ ^21^ ^]^ Finally, the dual‐side‐patterned AEM was gently removed from the meshes, and the MEA for the AEMWE was constructed by spraying a commercially available IrO_2_‐containing slurry for the anode and a Pt/C‐containing slurry for the cathode. The effects of the dual‐sided microstructured AEM in the MEA for AEMWE are schematically depicted in Figure 1b. The enlarged membrane‐electrode interfacial area, resulting from the formation of a 3D‐interlocked structure, enhanced the interfacial adhesion strength, which is a crucial factor in the durability of the AEMWE.^[^ ^5^, ^12^, ^16^ ^]^ Furthermore, the creation of additional hydroxide ion transport pathway at the interface further benefits performance. The overall thickness of the AEM decreases, and this modification creates locally shorter ion conduction pathways, enhancing the movement of hydroxide ions from the cathode to the anode.^[^ ^13^, ^22^ ^]^ Simultaneously, water, which serves as a reactant for the cathode reaction, was transported through back‐diffusion from the anode to the cathode. In pure water‐fed AEMWE conditions without a supporting electrolyte, electrochemical reactions occur exclusively at the TPBs located at sites containing electron conductors, active catalysts, anionic carriers, and pathways for reactants and products.^[^ ^5a^ ^]^ Increasing the ionomer content in the electrode might seem beneficial, but it can severely reduce mass transfer and catalyst utilization.^[^ ^23^ ^]^ Therefore, expanding the membrane/electrode interface without adding more ionomers to the electrode helped secure more TPB by increasing the number of hydroxide ion pathways.

### 2.2 Enhanced Pattern Fidelity by Optimizing Manufacturing Factors in the Patterning Process

**Figure 2:**
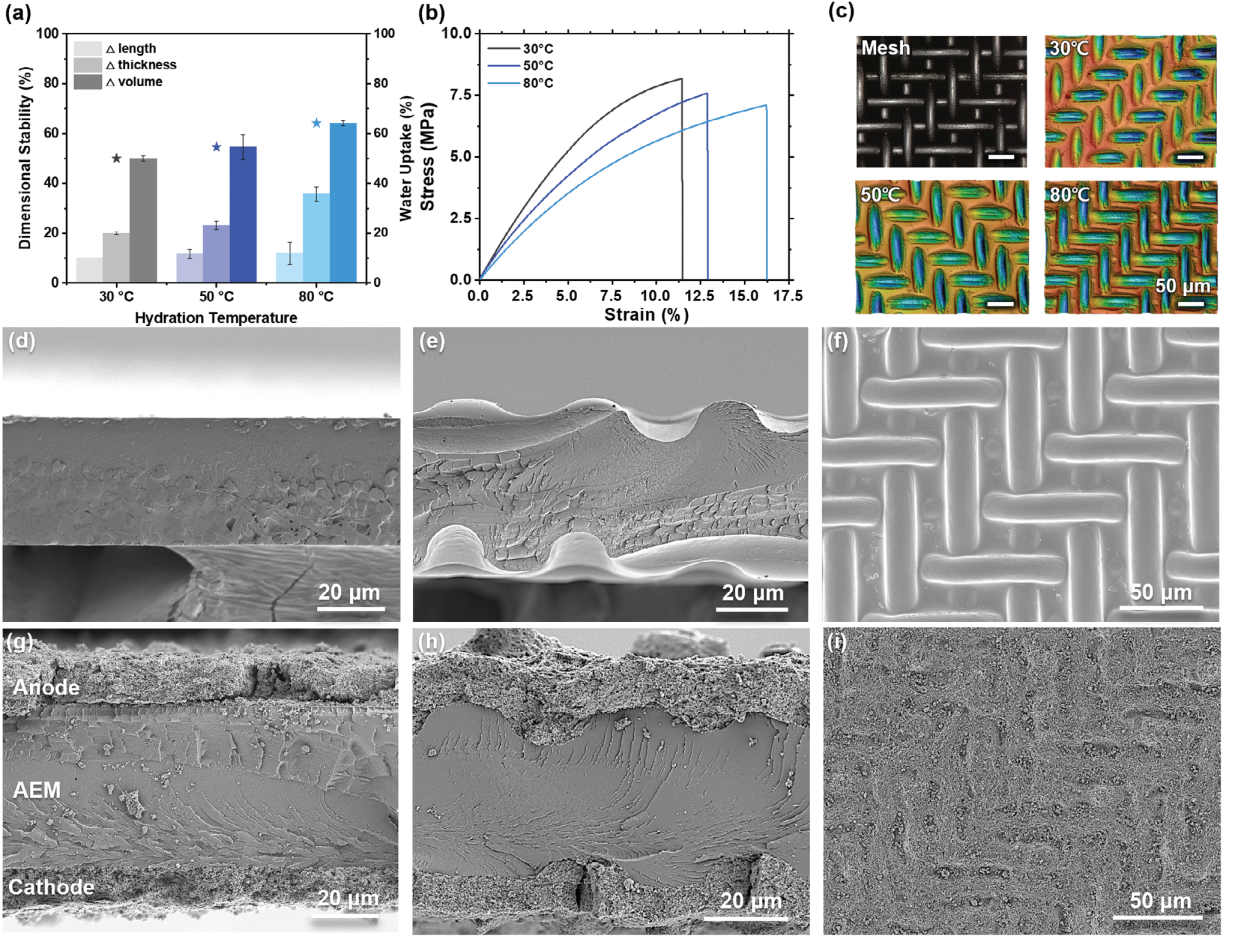
a) Dimensional changes and water uptake, and b) stress‐strain curves of the AEMs depending on different hydration temperature conditions. c) Optical image of SUS316L mesh and 3D laser profiled images of patterned AEMs with different hydration conditions. Cross‐sectional SEM images of d) pristine AEM and e) dual‐side patterned AEM. f) Surface SEM image of patterned AEM. Cross‐sectional SEM images of MEAs with g) pristine AEM and h) dual‐side patterned AEM. i) Surface SEM image of MEA with patterned AEM.

**Figures** **2**a and S1 (Supporting Information) illustrate the dimensional changes in the length and thickness of the AEM in response to various hydration conditions. After 24 h hydration, the water uptakes of PiperION membrane were 50% at 30 °C, 53.6% at 50 °C, and 64.2% at 80 °C. AEM exhibited the lowest dimensional change of 10% in length and 20% in thickness direction when subjected to 30 °C hydration for 24 h. On the other hand, while no significant differences were observed in length (in‐plane direction), a substantial difference was evident in the thickness direction, with the AEM undergoing a 35.8% change in thickness under 80 °C hydration conditions. Although temperature dependent, commercial PiperION AEM with a high ionic exchange capacity (IEC) of ≈ 2.35 exhibits a high water absorption capacity, particularly at 80 °C. This high water uptake promotes plasticization of the AEM, and a large dimensional change in the thickness direction is provided for spatial deformation for pattern formation. The stress‐strain curve of the dry PiperION AEM is shown in Figure S2 (Supporting Information). The pristine PiperION AEM exhibited a high Young's modulus of 981 MPa and a tensile strength of 45.7 MPa, indicating that its mechanical deformation of AEM was challenging owing to its high rigidity. However, as the membrane swells due to hydration, its mechanical properties undergo notable changes, as illustrated in Figure 2b. The Young's modulus decreases from 124 MPa to 89.5 MPa as the hydration temperature rises from 30 to 80 °C, while tensile strength decreases from 8.18 MPa to 7.11 MPa in a parallel manner. In conjunction with the aforementioned plasticized AEM, which exhibits severely reduced mechanical rigidity, metal meshes manufactured from SUS316L, which has a high tensile strength of 176 GPa^[^ ^24^ ^]^ can be utilized for molding purposes by inducing creep deformation in the AEM. The meshes had a wire diameter and spacing distance of 20 µm each and an opening ratio of 25%, as illustrated in Figures 2c and S3 (Supporting Information). Thin metal meshes were supplied in a roll‐type configuration, as depicted in Figure S4 (Supporting Information). While a basic mesh size of 300 mm × 500 mm was initially utilized for membrane patterning, our patterning technique allows for large‐scale production, including roll‐to‐roll processes, without requiring complex patterning techniques or expensive molds. The patterned AEMs manufactured under various hydration conditions using the optimized patterning process (dehydration at 80 °C for 2 h with 10 MPa pressure; see Note S1 and Figures S5 and S6, Supporting Information) are shown in the 3D laser profile images (Figure 2c; Figure S7, Supporting Information). The shape varies significantly with the hydration state of the membrane. The mold's shape is accurately reproduced in the 80 °C AEM, which exhibits significant water absorption and high plasticity. In contrast, the cross‐sectional scanning electron microscope (SEM) images demonstrate that the pattern depth at 30 and 50 °C does not correspond to the depth of the mesh mold as depicted in Figure S8 (Supporting Information). At 80 °C hydration conditions, the AEM underwent significant dimensional changes in the thickness direction, resulting in the formation of a mesh pattern on both the top and bottom sides of the membrane. Similar to the hydration conditions, the patterning pressure and temperature were also found to have a significant impact on pattern fidelity, with the optimal pattern fidelity finally achieved at a patterning condition of 10 MPa pressure and 80 °C (see Note S1, Supporting Information). The original mechanical properties were restored by dehydration after creep deformation was induced in the plasticized membrane (Figure S2, Supporting Information). Consequently, the objective of minimizing immediate form restitution by creep recovery was achieved by removing the mesh molds.

### 2.3 Characterizations of the Patterned Membrane

The AEM is characterized by a flat surface with a thickness of 40 µm (Figure 2d). In contrast, the dual‐side patterned AEM, as illustrated in Figure 2e,f, exhibits a grooved area with a depth of ≈7.4 µm, resulting from wire‐engraving with mesh molds. The fixed constraints precluded restoration of the hydrated film in the in‐plane direction after dehydration, resulting in an average total film thickness of 38.7 µm. Following the patterning process, the mechanical characteristics of the dual‐sided patterned AEM at 80 °C were comparable to those of the pristine AEM under dry conditions (Figure S2, Supporting Information). Additionally, the dimensional change and water uptake, reflecting the swelling characteristics, were nearly identical to those of pristine AEM (Table S1, Supporting Information). The pristine AEM and patterned AEM exhibited nearly equal in‐plane conductivities of 111 and 116 mS cm^−1^, respectively, at 45 °C in terms of ion‐conducting characteristics (Table S2, Supporting Information). This indicates that the eco‐friendly patterning method developed in this study employed only water and metal mesh molds, thereby preserving the mechanical and ion‐conducting capabilities of the membrane. The electrodes in the MEA with the patterned membrane were well formed along the grooved surface of the patterned membrane, creating a 3‐D interlocked structure using the CCM method by directly spraying the catalyst slurries, as depicted in Figure 2h,i. This is in contrast to the flat 2D interfaces of the MEA with the pristine membrane shown in Figure 2g.

### 2.4 Evaluation Patterned AEMWE with Commercial Membrane under Different Supporting Electrolyte Conditions

**Figure 3:**
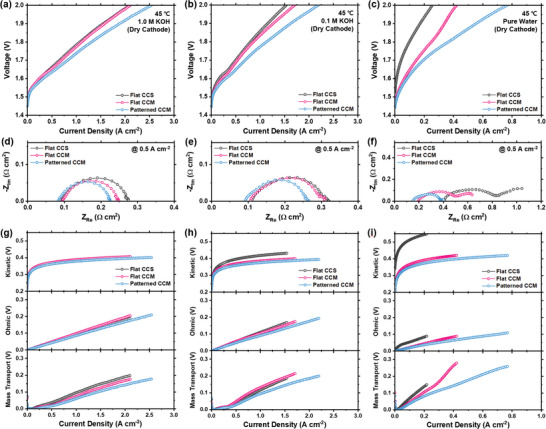
a–c) Polarization curves of different MEAs in a) 1.0 m KOH, b) 0.1 m KOH, and c) pure water at a cell temperature of 45°C. d–f) Nyquist plots of total cell impedance measured at 0.5 A cm^−2^ of different MEAs in d) 1.0 m KOH, e) 0.1 m KOH, and f) pure water at 45°C. g–i) Breakdown of overpotentials (kinetic, ohmic, and mass transport) for different MEAs in g) 1.0 m KOH, h) 0.1 m KOH, and i) pure water at a cell temperature of 45 °C. MEAs were fabricated with an IrO_2_/PiperION catalyst layer with Ti felt (anode), Pt/C/PiperION catalyst layer with carbon‐paper PTL (cathode), and 40 µm PiperION membrane.

To confirm the impact of the 3‐D interlocked membrane‐electrode interface on the performance of the AEMWE, the validation step for the patterning method was initially carried out using the commercial PiperION AEM. Two MEAs (Flat CCM and Patterned CCM) were fabricated using the CCM method with two types of electrolyte membranes (flat reference and mesh‐patterned membranes). Additionally, an MEA fabricated by the CCS method utilizing a flat reference membrane (Flat CCS) was fabricated to examine the impact of interfacial contact properties on AEMWE performance. Generally, the contact between the catalyst layer and the membrane is inferior in CCS MEAs compared to CCM MEAs. Therefore, comparing the electrochemical characteristics of Flat CCS and Flat CCM serves as a useful reference for analyzing the characteristics of the Patterned CCM with a 3‐D interlocked membrane‐electrode interface. **Figure** **3**a–c shows the results of evaluating the water electrolysis performance of the three MEAs under different supporting electrolyte conditions (1.0 m KOH, 0.1 m KOH, and pure water) at a temperature of 45 °C. While both MEAs show similar performance in 1.0 m KOH fed conditions (1.585 A cm^−2^ at 1.9 V for Flat CCM and 1.551 A cm^−2^ at 1.9 V for Flat CCS), the performance of Flat CCM (0.338 A cm^−2^ at 1.9 V) was nearly double that of the Flat CCS (0.172 A cm^−2^ at 1.9 V) in pure water fed condition. This stark contrast demonstrates that ion conduction at the membrane‐electrode interface is a key factor in cell performance under pure water‐fed conditions without a supporting electrolyte, indicating that ensuring effective ion pathways within the cell is crucial for pure water‐fed AEMWE. As illustrated in Figure 2h, the catalyst layers of the Patterned CCM were well covered, with no defects along the patterned boundaries. The layers possessed an extended contact interface compared to the flat interface. Furthermore, the catalyst layer formed along the groove structure creates shorter and more ionic pathways across the electrode, potentially ensuring more efficient ion transport within the cell. Consequently, the Patterned CCM outperformed the other two Flat MEAs under all test conditions, particularly in the pure water‐fed condition without any supporting electrolytes. For example, the Patterned CCM shows only a 19.0% improvement over the Flat CCM under 1.0 M KOH fed condition (1.886 A cm^−2^ for Patterned CCM and 1.585 A cm^−2^ for Flat CCM), but a 63.9% improvement under pure water fed condition (0.563 A cm^−2^ for Patterned CCM and 0.338 A cm^−2^ for Flat CCM). This significant performance improvement under pure feed conditions was also observed across different operating temperatures of 45, 60, and 80 °C (Figure S9a–c, Supporting Information). However, as the temperature increases, the performance gap between the Flat and Patterned CCMs narrows. This trend can be attributed to the temperature‐dependent nature of ion transport in AEMWE. At lower temperatures (e.g., 45 °C), the limited ion mobility makes the enhancements provided by the Patterned CCM—such as expanded interfacial areas and improved hydroxide ion transport—particularly significant. Nevertheless, the Patterned CCM still demonstrates superior performance, showing ≈ 20.5% improvement even at an operating temperature of 80 °C at 1.9 V (Figure S9d, Supporting Information). Moreover, when compared with Flat CCM containing 20% anode ionomer (i.e., a twofold increase in ionomer usage), it demonstrated comparable performance under pure water conditions compared to that of Patterned CCM. However, the increased ionomer content resulted in the poorest performance under 1.0 m and 0.1 M KOH conditions in comparison to the reference Flat and Patterned CCM, as illustrated in Figure S10 (Supporting Information). The drop in performance indicates that in environments that have sufficient supporting electrolyte, excess ionomer may block active sites or obstruct mass transport due to pore blockage. This implies that the Patterned CCM design facilitates efficient ion transport with minimal ionomer while mitigating the performance drawbacks associated with raised ionomer content, thus offering a more effective and resilient solution for both pure water‐fed AEMWE and conventional AEMWE applications. Additionally, when patterning was conducted under suboptimal conditions, such as at 30 and 50 °C, the membrane showed low pattern fidelity, which led to a notable decline in overall performance and an increase in both ohmic and charge transfer resistance (Figure S11, Supporting Information). Therefore, is essential to ensure high pattern fidelity to facilitate proper 3D interlocking between membrane and electrode, which is critical for enhanced ion transport and cell performance.

**Figure 4:**
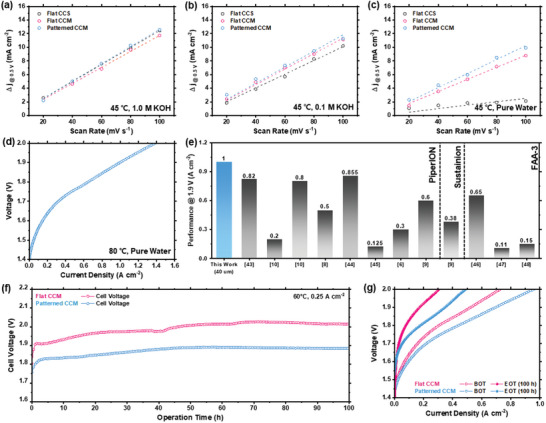
EDLC of the anode for different MEAs in a) 1.0 m KOH, b) 0.1 m KOH, and c) pure water at a cell temperature of 45°C. d) Polarization curves of pure‐water‐fed AEMWE performance of Patterned CCM cell at a temperature of 80 °C e) Comparison of the performance of pure water‐fed AEMWEs using commercial AEMs.^[^ ^3^, ^24^ ^]^ f) Long‐term durability of pure water‐fed AEMWEs operated at 0.25 A cm^−2^ and 60 °C for 100 h. g) Polarization curves of pure water‐fed AEMWEs before and after durability test at 60 °C.

The superiority of the Patterned CCM to other MEAs is also evident from the electrochemical impedance spectroscopy (EIS) spectra measured at 0.5 A cm^−2^. Figure 3d–f shows that the Patterned CCM cell exhibited a lower high‐frequency resistance (HFR) than the other two cells under all test conditions. This result demonstrates that the enlarged interfacial area owing to the patterned surface improves the adhesion between the electrode and membrane, thus successfully reducing the interfacial contact resistance. This suggests that ion transport at the 3D interlocked membrane‐electrode interface was more efficient than that at the flat structure. Although MEAs with a flat reference membrane exhibited similar HFRs under 1.0 m KOH fed conditions, the Flat CCS (0.406 Ω cm^2^) exhibited nearly double the HFR compared to the Flat CCM (0.195 Ω cm^2^) under pure water‐fed conditions. This significant difference in the HFR between the two MEAs utilizing the same electrolyte membrane underscores the critical role of the membrane‐electrode interface design in achieving high‐performance pure water‐fed AEMWE systems. Furthermore, the Patterned CCM cell exhibited the lowest charge transport resistance regardless of the electrolyte feed conditions, providing further insight into the effectiveness of the 3D interlocked interface design in improving electrode performance. In particular, the pure water‐fed Flat CCS and Flat CCM cells exhibited distinctly large low‐frequency semicircles related to mass‐transport resistance, whereas the pure water‐fed Pattern CCM cell exhibited a significantly smaller low‐frequency semicircle. These results indicate that the Patterned CCMs facilitated ion transport not only at the membrane‐electrode interface but also within the electrode by providing shorter and additional ion pathways across the electrode due to its grooved structure (Figure 1b). This improved ion accessibility allowed the patterned electrode to maintain a high electrochemically active surface area (ECSA) even in environments with insufficient supporting electrolytes, and the local load of each active point is reduced, resulting in improved electrode mass transfer. This is supported by the finding that the Warburg impedance increases with decreasing pattern fidelity (Figure S10, Supporting Information). Cyclic voltammetry was conducted over a voltage range of 0.4 to 0.6 V at scan rates of 20, 40, 60, 80, and 100 mV s^−1^ for the different MEAs with various electrolyte feed conditions (Figure S12, Supporting Information). As shown in **Figure** **4**a–c, the anode for the Patterned CCM not only exhibited the highest electrochemical double layer capacitance (EDLC) across all electrolyte feed conditions but also retained a higher EDLC in the pure water‐fed condition compared to the 1.0 m KOH‐fed condition. Specifically, the anode of the Patterned CCM retained 81.7% of its EDLC, whereas those of the Flat CCM and Flat CCS retained 75.2% and 20.0%, respectively. This suggests that the patterned interface provides sufficient ionic pathways without any supporting electrolyte, supporting a more uniform reaction rate across the electrode.

To better understand the impact of modifying the interfacial design on cell performance, the overpotential of the cell was separated into three components: kinetic overpotential, ohmic overpotential, and mass transport overpotential. Figure 3g–i demonstrate that the Patterned CCMs consistently showed lower kinetic, ohmic, and mass transport overpotentials than both the Flat CCS and Flat CCMs, regardless of the test conditions. The disparity in overpotentials among the tested MEAs was most evident under pure water‐fed conditions without any supporting electrolytes. For example, the kinetic overpotentials related to the electrochemical processes at the electrode surface were reduced for the Patterned CCM compared to the Flat CCM, indicating improved utilization of the active sites offered by the patterned structure. Additionally, the Flat CCS showed a significantly higher kinetic overpotential compared to the MEAs with the CCM interface owing to inferior membrane‐electrode contact. In addition, the ohmic overpotential, which is associated with the resistance to ion transport in the cell, was minimized in the Patterned CCM due to the improved ionic conductivity resulting from the optimized ion transport pathways in the patterned structure. Moreover, similar to the electrochemical impedance results (Figure 3f), the Patterned CCM demonstrated a notably reduced mass transport overpotential compared to the other two cells. This result indicates that the patterned‐electrode design effectively resolved the mass‐transport‐related limitations experienced by the other two electrodes.

### 2.5 Performance and Durability Test of Pure Water‐Fed AEMWE using the Patterned Commercial Membrane

Given the compelling characteristics of Patterned CCM under pure water‐fed conditions, we further evaluated its performance at an elevated temperature of 80 °C. As shown in Figure 4d, the pure water‐fed Patterned CCM cell demonstrated a current density of ≈ 1.0 A cm^−2^ at 1.9 V, outperforming most pure water‐fed AEMWEs using commercial AEMs (ex. PiperION, Sustainion, and FAA‐3) (Figure 4e).^[^ ^3^, ^25^ ^]^ Detailed comparisons, including the AEM thickness, anode catalyst, cathode catalyst, ionomer, porous transport layer, operating temperature, and feeding conditions, are summarized in Table S3 (Supporting Information).  This result emphasizes the notable enhancement in the pure water‐fed AEMWE performance, which can be accomplished by changing the interfacial design without requiring any specialized materials.

In situ durability tests were conducted to investigate the impact of the patterned interfacial structure on the durability of the pure water‐fed AEMWEs. The Patterned CCM maintained a lower cell voltage compared to the Flat CCM for over a 100 h of operation at 60 °C and 0.25 A cm⁻^2^ (Figure 4f). In addition, the Patterned CCM showed a slower increase in cell voltage (1.28 mV h⁻^1^) over time compared to the Flat CCM (2.1 mV h⁻^1^), indicating better stability of the Patterned CCM. This indicates that the 3D interlocking structure of the Patterned CCM is favorable for maintaining membrane‐electrode contact. Therefore, the Patterned CCM retained a better performance than the flat CCMs, even after the durability test (Figure 4g). The enhanced durability of the Patterned CCMs was attributed to the improved structural integrity provided by the 3‐D interlocked interface design. Specifically, enhanced interfacial adhesion and increased active site utilization play crucial roles in maintaining long‐term stability, thereby ensuring consistent performance during extended periods of operation.

As shown in Figure S13 (Supporting Information), the post‐mortem analysis of cross‐sectional SEM images after the durability test revealed that the loss of the anode catalyst layer from the membrane was significantly reduced in the Patterned CCM compared to the Flat CCM. The improved interfacial adhesion was quantitatively measured through a mechanical interfacial adhesion test. Figure S14a (Supporting Information) shows the maximum shear stress of the prepared laminates. While the flat CCM laminate exhibited a maximum shear stress of ≈46.2 kPa, the patterned CCM laminate displayed a remarkably higher shear stress of 95.0 kPa, ≈105.6% greater than that of the flat configuration, indicating significantly stronger adhesion at the membrane‐electrode interface. Additionally, as shown in Figure S14b (Supporting Information), digital images of the CCM samples after the shear stress test revealed that the patterned membrane retained much of the electrode material on its surface, whereas the flat membrane exhibited substantial electrode detachment, exposing the bare membrane. The corresponding surface and cross‐sectional SEM images further confirmed the improved structural integrity of the Patterned CCM. Consequently, it was demonstrated that the 3D interlocked structure not only enhanced the initial performance but also provided better durability by maintaining effective ionic conduction and membrane‐electrode contact over extended operation periods. These findings indicate that modifying the membrane‐electrode interface structure is a promising strategy for future AEMWE applications, particularly under pure water‐fed conditions.

### 2.6 Universal and Practical Applicability of Patterning Process

**Figure 5:**
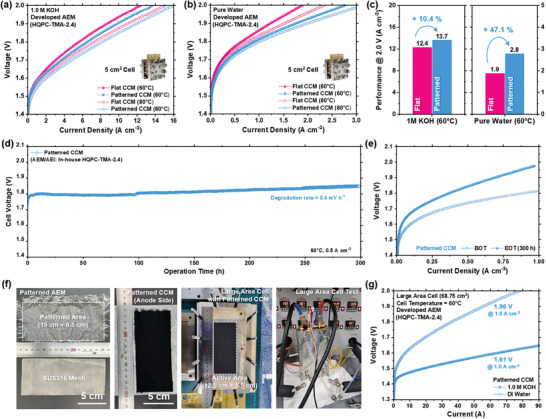
Polarization curves for 5 cm^2^ cells using flat and patterned CCMs in a) 1.0 m KOH‐fed (both feed), and b) pure water‐fed condition (dry cathode). The inset shows the actual 5 cm^2^ cell setup. c) Performance comparison at 2.0 V for flat and patterned CCMs in 1.0 m KOH‐fed and pure water‐fed conditions. d) Long‐term durability of pure water‐fed AEMWE with patterned CCM operated at 0.5 A cm^−2^ and 60 °C for 300 h. e) Polarization curves of pure water‐fed AEMWE with patterned CCM before and after durability test at 60 °C. f) Images of the patterned AEM, CCM, and single‐cell setup for large‐size cell with an active area of 68.75 cm^2^ g) Polarization curves of large‐size cell with patterned CCM in 1.0 m KOH‐fed and pure water‐fed conditions. MEAs were fabricated with an IrO_2_/HQPC‐TMA‐2.4 catalyst layer with SUS316 felt (anode), PtRu/C/HQPC‐TMA‐2.4 catalyst layer with carbon‐paper (cathode), and 30 µm HQPC‐TMA‐2.4 membrane.

To verify the versatility of the developed patterning method, the performances of the MEAs with Flat CCM and Patterned CCM were further evaluated using in‐house developed anion exchange membranes and ionomers (Figure S15, Supporting Information). As anion‐conducting material, we selected the HQPC‐TMA‐2.4, one of the leading‐edge anion‐conducting polymers that demonstrated superior performance under full‐cell test conditions.^[^ ^26^ ^]^ Specifically, the HQPC‐TMA‐2.4 ionomer demonstrated superior pure water‐fed AEMWE performance due to its higher volumetric ion exchange capacity (IECv) compared to commercial PiperION. This enhanced IECv means that HQPC‐TMA‐2.4 incorporates more ion exchange groups within the same volume, which leads to improved ion conduction pathways across the membrane and electrode. **Figure** **5**a,b shows the results of evaluating the AEMWE performance of the two different cells under different electrolyte feed conditions (1.0 m KOH and pure water), confirming that the patterned membrane‐electrode interface was beneficial for AEMWE performance, regardless of the anion conducting material used. In addition, EIS analysis showed that the Patterned CCMs exhibited lower HFR and charge transport resistance compared to the Flat CCMs, especially under pure water‐fed condition. (Figure S16, Supporting Information). The greater performance enhancement of the Patterned CCM in the absence of a supporting electrolyte underscores the effectiveness of the interlocking between the membrane‐electrode via patterning in facilitating ion transport and reducing polarization loss in a cell. Specifically, at 60 °C and 2.0 V, the HQPC‐TMA‐2.4‐based Patterned CCMs achieved 13.7 A cm^−2^ in 1.0 m KOH and 2.8 A cm^−2^ in pure water, representing a 10.4% and 47.1% improvement over the Flat CCMs, respectively (Figure 5c). Furthermore, at 80 °C, the HQPC‐TMA‐2.4‐based Patterned CCMs still exhibit 33% higher performance, achieving 3.2 A cm^−^^2^ under pure water‐fed conditions (Figure S17, Supporting Information). Moreover, the in‐situ durability test at 0.5 A cm^−2^ and 60 °C indicates that the degradation rate of our patterned membrane is significantly low compared to other state‐of‐the‐art AEMWEs under similar conditions. (Figure S18, Supporting Information)^[^ ^3^, ^4^, ^6^, ^12^, ^16^, ^25^, ^26^, ^27^ ^]^ Specifically, the patterned HQPC‐TMA‐2.4 membrane maintained stable performance with a minimal degradation rate of 0.4 mV h⁻^1^ over 300 h, highlighting its robustness in pure water‐fed applications.

To further validate the scalability of the patterning process and their practical applicability in real‐world application, the large‐size cell with an active area of 68.75 cm^2^ was fabricated and evaluated using the patterned HQPC‐TMA‐2.4 membrane. Figure 5d shows digital camera images of the patterned membrane and CCM, and the single cell setup for the large‐size cell test. Despite using the thin 30 µm membrane, the patterned large‐area membrane was successfully fabricated (Figure S19, Supporting Information) and integrated into the large‐size cell as an ionic conductor and an electrical insulator without any pinhole formation. Consequently, the large‐size cell with the patterned CCM achieved 1.61 and 1.96 V at a current density of 1.0 A cm⁻^2^ under 1.0 m KOH and pure water‐fed condtion, respectively. While the area‐specific performance of the large‐size cell was lower than that of the small‐size cell (Figure S20, Supporting Information) due to several factors like increased ohmic resistance, non‐uniform current distribution, and potential issues with water management and gas bubble removal over large areas,^28^ ^]^ it is noteworthy that the large‐size cell with patterned HQPC‐TMA‐2.4 membrane still exhibited superior area‐specific performance of 0.81 A cm^−2^ at 1.9 V compared to most of commercial AEM‐based pure water‐fed AEMWEs (Figure 4e).

## 3 Conclusion

In summary, a straightforward patterning method was developed to modify the surface morphology of hydrocarbon‐based AEM with the aim of enhancing the efficiency of AEMWE. This method increases the membrane‐electrode interface area and shortens the ion conduction pathway within the cell. This environmentally friendly approach employs water for hydration‐induced swelling and plasticizes the AEM in conjunction with metal meshes to generate mechanical creep deformation in the AEM. This was achieved by exploiting the high water absorption properties and changes in the thickness of the AEM. The dehydration process, conducted under fixed constraints, regained the original mechanical properties of the patterned AEM. This resulted in high pattern fidelity by alleviating the instant shape recovery upon the removal of the mechanical load. A single‐cell test demonstrated that the use of the patterned membrane significantly enhanced the performance of the AEMWE compared with conventional AEMWEs with flat membranes, regardless of the AEM used (commercial PiperION and in‐house fabricated HQPC‐TMA‐2.4). Specifically, the AEMWE with the patterned HQPC‐TMA‐2.4 membrane reached an excellent current densities of 13.7 A cm^−2^ at 2.0 V in 1.0 m KOH, and 2.8 A cm^−2^ at 2.0 V in pure water, which are comparable to those of the state‐of‐the‐art AEMWEs, despite the low operating temperature of 60 °C. In addition, in situ durability tests revealed that the use of the patterned membrane not only enhanced the initial performance but also provided better durability. This improvement is attributed to the extended ionic network at the membrane‐electrode interface and the localized reduction of the ion transport pathway through the AEM, which reinforces interfacial adhesion and mitigate local pH reduction. Furthermore, the scalability of the technique was further demonstrated through successful large‐area cell fabrication and operation. These findings highlight the proposed patterning technique is versatile, beneficial for scaling‐up, and easily adaptable to various AEMs. This advancement is expected to significantly impact the evolution of AEMWE, particularly in the context of future neutral seawater electrolysis.

## Conflict of Interest

The authors declare no conflict of interest.

## Supporting information



Supporting Information

## Data Availability

The data that support the findings of this study are available from the corresponding author upon reasonable request.
