# Supplementary material for: High‐Performance Pure Water‐Fed Anion Exchange Membrane Water Electrolysis with Patterned Membrane via Mechanical Stress and Hydration‐Mediated Patterning Technique
Source: Adv Sci (Weinh). 2024 Dec 16;12(5):2409563. doi: 10.1002/advs.202409563 (PMC11792040; doi:10.1002/advs.202409563)
Supplement: Supplementary file 1 — Supporting Information [file ADVS-12-2409563-s001.pdf]

ADVANCED SCIENCE
Open Access

Supporting Information

for *Adv. Sci.*, DOI 10.1002/advs.202409563

High-Performance Pure Water-Fed Anion Exchange Membrane Water Electrolysis with Patterned Membrane via Mechanical Stress and Hydration-Mediated Patterning Technique

Yeonjae Lee, Sungjun Kim, Yoseph Shin, Yeram Shin, Seongmin Shin, Sanghyeok Lee, Minseop So, Tae-Ho Kim, Sehkyu Park, Jang Yong Lee and Segeun Jang**

Supporting Information for

High-performance pure water-fed anion exchange membrane water electrolysis with patterned membrane via mechanical stress and hydration-mediated patterning technique

Yeonjae Lee, Sungjun Kim, Yoseph Shin, Yeram Shin, Seongmin Shin, Sanghyeok Lee, Minseop So, Tae-Ho Kim, Sehkyu Park, Jang Yong Lee*, and Segeun Jang*

Y. Lee, Y. Shin, S. Lee, M. So, S. Jang

School of Mechanical Engineering, Kookmin University, Seoul 02707, Republic of Korea

E-mail: sjang@kookmin.ac.kr

S. Kim, Y. Shin, S. Shin, T.-H. Kim

Hydrogen Energy Research Center, Korea Research Institute of Chemical Technology (KRICT), Daejeon 34114, Republic of Korea

S. Park

Department of Chemical Engineering, Kwangwoon University, Seoul 01897, Republic of Korea

J. Y. Lee

Department of Chemical Engineering, Konkuk University, Seoul 05029, Republic of Korea

E-mail: ljyljy78@konkuk.ac.kr

Keywords: anion exchange membrane water electrolysis, pure water fed, patterned membrane, dehydration, enlarged interfacial area

Table of contents

Experimental Details **1**

Supplementary Note 1 .... **5**

Supplementary Figures **6**

Figure S1 6

Figure S2 7

Figure S3 8

Figure S4 9

Figure S5 10

Figure S6 11

Figure S7 12

Figure S8 13

Figure S9 14

Figure S10 15

Figure S11 16

Figure S12 17

Figure S13 18

Figure S14 19

Figure S15 20

Figure S16 21

Figure S17 22

Figure S18 23

Figure S19 24

Figure S20 25

Supplementary Tables **26**

Table S1 26

Table S2 27

Table S3 28

Experimental Details

Chemicals and materials

Alpha Aesar supplied iridium oxide (IrO_2, Premion 99.99% metal base) and Platinum, nominally 40%, Ruthenium, nominally 20% on carbon black (PtRu/C). Platinum on carbon with a platinum content of 46.9 wt% (Pt/C) was obtained from Tanaka Kikinzoku Kyogyo (TKK). The commercial PAP-TP-85 membranes (40 μm) and 5 wt% ionomer dispersions in ethanol were purchased from Versogen, Inc. The in-house developed HQPC-TMA-2.4 membranes (30 μm) and 5 wt% ionomer dispersions were prepared based on a previously published paper.[1] Daeyoung Sigma Filter Inc. supplied SUS-316L mesh with a wire diameter and spacing distance of 20 μm each. The Pt-coated titanium felt (Currento® PTL Ti-56/250 PT200) and SUS316 felt (10 AL3) was acquired from Bekaert Inc. The microporous layer coated carbon papers (Sigracet 39BB and SGL-39BC) was obtained from SGL Carbon Inc. Samchun Chemical was the source of the isopropyl alcohol (99.5%) and potassium hydroxide (85.0%) utilized in this study.

Synthesis of in-house anion conducting polymer (HQPC-TMA-2.4)

9-(6-bromohexyl)-9H-carbazole (BHC) monomer and the chain extender 1,6-di(9H-carbazol-9-yl)hexane (DCH) were synthesized as detailed in prior work.[1] For the production of a high-molecular-weight polymer backbone, BHC (111.50 g, 337.60 mmol) was introduced into a flame-dried 1 L three-neck flask fitted with an overhead mechanical stirrer and dissolved in dichloromethane. The solution was subjected to cooling in an ice bath (0 °C) for 30 minutes and thereafter augmented with 1,1,1-trifluoroacetone (51.07 g, 455.76 mmol) and trifluoromethanesulfonic acid (TFSA, 500 g, 3332.22 mmol) while maintaining continuous agitation. Subsequent to the incorporation of TFSA, the solution was chilled for three hours and permitted to react at room temperature. After 24 hours, DCH (0.56 g, 1.34 mmol) was introduced through syringe injection, resulting in a thick dark brown liquid following a further 48 hours of reaction. The resultant mixture was precipitated in fresh methanol, and the fibrous product was filtered, washed with hot methanol, and dried at 80 °C in a vacuum oven for 24 hours to yield high-molecular-weight poly(9-(6-bromohexyl)-9H-carbazole-co-1,1,1-trifluoroisopropane-co-9-hexyl-9H-carbazole) (HPC-br-x). HPC-br-x was introduced into a three-neck flask and solubilized in N,N-dimethylformamide. The solution was gradually augmented with 45% aqueous trimethylamine and agitated at room temperature for 24 hours. The product was precipitated with fresh isopropanol, subjected to multiple washes, and dried under vacuum at 60 °C for 24 hours to get quaternized high-molecular-weight poly(9-(6-(trimethylammonium bromide)hexyl)-9H-carbazole-co-1,1,1-trifluoroisopropane-co-9-hexyl-9H-carbazole with ion exchange capacity of 2.4 (HQPC-TMA-2.4).

Preparation of in-house membrane and ionomer solution

HQPC-TMA-2.4 membrane with thicknesses of 30 μm was fabricated using a conventional solvent casting technique. HQPC-TMA-2.4 powder was solubilized in dimethyl sulfoxide at a concentration of 20 wt%. The

solution was filtered using a syringe fitted with a 5 μm polytetrafluoroethylene (PTFE) membrane filter, deposited onto a glass plate, and dried at 80 °C for 4 hours. Upon drying, the membrane was removed from the glass plate by immersion in 1.0 M KOH and subsequently rinsed or immersed in deionized water for several minutes to remove any residual KOH. The ionomer solution was formulated by dispersing the synthesized HQPC-TMA-x in an H_2O:n-propanol mixture (6:4, w/w) at room temperature with a loading of 5 wt%.

Preparation of patterned membrane and the MEAs

The membranes (commercial 40 μm PiperION® (Versogen, USA), or in-house fabricated 30 μm HQPC-TMA-2.4) were treated with 1M KOH for 3 hours, with the solution being refreshed every 30 minutes. For the preparation of the patterned membrane, the KOH-treated membrane was soaked in distilled water at 80°C for 24 hours. The swollen and hydrated membrane was inserted between two SUS-316L meshes. A pressure of 10MPa was applied to the sandwiched assembly of the membrane and the meshes until the membrane was sufficiently dehydrated. Following this, the patterned membrane was carefully peeled off from the meshes. To construct the MEA with the CCM method, an anode catalyst slurry was prepared with iridium oxide (IrO_2), deionized water, isopropyl alcohol, and anion exchange ionomers (commercial 5 wt.% PiperION® dispersion, or in-house fabricated 5 wt.% HQPC-TMA-2.4 dispersion). A cathode catalyst slurry was also prepared with the same components as the anode slurry, but replacing IrO_2 with Pt/C (for PiperION®-based cathode) or PtRu/C (for HQPC-TMA-2.4-based cathode). For PiperION-based electrodes, the ionomer to catalyst weight ratios were 10 wt.% for the IrO_2 anode and 50 wt.% for the cathode. For in-house fabricated HQPC-TMA-2.4-based electrodes, the ionomer to catalyst weight ratios were 20 wt.% for the IrO_2 anode and 20 wt.% the cathode. The prepared solutions were sprayed onto the membrane using a stainless-steel mask with a square hole of 2.23 × 2.24 cm (width by height, active area of 5 cm^2) until reaching the target platinum group metal (PGM) loading of 2.0 mg cm^{-2} for anode and 0.4 mg cm^{-2} for cathode, respectively. For purpose of comparison, the MEA with CCS method was prepared by spraying the catalyst slurries onto the porous transport layer with the same loading amount.

Measurement of the AEMWE performance

A single-cell assembly was utilized to assess the performance of AEMWE. The cell hardware consisted of sets of SUS-316L end plates, Au-coated current collectors, and Ni-coated SUS-316L flow plates with a single serpentine channel with a 1mm width and height. For PiperION-based cell, the prepared CCM was placed between the anode Pt-coated Ti felt and the cathode carbon paper (SGL-39BB) with two PTFE-film gaskets (240 μm for the anode and 200 μm for the cathode) and tightly assembled with single-cell components. For HQPC-TMA-based cells, the fabricated CCM was sandwiched between the anode SUS-316 felt and cathode carbon paper (SGL39BC) with two PTFE-film gaskets (270 μm for the anode and 220 μm for the cathode). The obtained single cells were integrated into an AEMWE test station (CNL Energy), and the electrochemical

investigations were conducted using a potentiostat (HCP-803; Bio-Logic). Prior to conducting the performance evaluation, a solution of 1.0 M KOH was introduced into the cell at a temperature of 45 °C with a flow rate of 10 mL min^{-1} for 1 hour. This was done to facilitate the exchange of hydroxide ions in the membrane and ionomers. Following preconditioning, the performance of the AEMWE was measured at different operating temperatures of 45 °C, 60 °C, and 80 °C. The measurement was conducted under asymmetric conditions of dry cathode and the anode supplied by the different KOH concentrations of 1.0, 0.1, and 0.0 M (pure water) with a flow rate of 5 ml min^{-1}. The I-V polarization curves were recorded through the linear sweep voltammetry (LSV) measurement within the voltage range of 1.3–2.0 V with a scan rate of 5 mV s^{-1}. Corresponding electrochemical impedance spectroscopy (EIS) measurements were performed at 0.5 A cm^{-2} under frequencies ranging from 0.1 Hz to 100 kHz with amplitudes of 40 mA cm^{-2}. The electrochemical active surface areas (ECSA) were determined using double-layer capacitance from cyclic voltammetry (CV) curves. The CV curves were obtained at 45°C under a potential range of 0.4 V to 0.6 V with various scan rates of 20, 40, 60, 80, and 100 mV s^{-1}. The durability test was performed under asymmetric (dry cathode) pure water-fed conditions with a constant current density of 0.1 A cm^{-2} and a temperature of 45 °C for 100 hours. During the durability test, corresponding high-frequency resistance (HFR) spectra were recorded using potentiostat. For large-size cell with an active area of 68.75 cm^2, the fabricated HQPC-TMA-2.4-based large-are CCM was sandwiched between the anode SUS-316 felt (12.5 × 5.5 cm) and cathode carbon paper (SGL39BC; 12.5 × 5.5 cm) with two PTFE-film gaskets (260 µm for the anode and 220 µm for the cathode). The obtained single cells were integrated into an AEMWE test station (CNL Energy), and the electrochemical investigations were conducted using a potentiostat (HCP-803; Bio-Logic). Prior to the performance evaluation, a solution of 1.0 M KOH was supplied to the cell at a temperature of 60 °C with a flow rate of 100 mL min^{-1} for 1 hour. Following the preconditioning step, the performance of the AEMWE was measured at an operating temperature of 60 °C. The measurement was conducted under asymmetric conditions of dry cathode and the anode supplied by the different electrolyte solution (1.0 M KOH and pure water) with a flow rate of 100 ml min^{-1}. The I-V polarization curves were recorded through the linear sweep voltammetry (LSV) measurement within the voltage range of 1.3–2.0 V with a scan rate of 2 mV s^{-1}.

Pure Water Flushing

Before measuring the AEMWE performance under pure water, a thorough flushing step with deionized water flowing at a rate of 20 ml min^{-1} for small-size cell and 100 ml min^{-1} for large-size cell over 2 hours was performed to eliminate any remaining KOH. The complete removal of alkaline residues was verified by monitoring the pH of the effluent from the vent line to verify it reached neutral levels.

Membrane Characterizations

Field-emission scanning electron microscopy (FE-SEM; SU-5000, HITACHI, Japan.) and the 3D laser profiler (VK-250K/260K, KEYENCE) were employed to observe morphological characteristics of the mesh, patterned

membrane, and MEAs. The stress-strain behavior of the membranes was measured using a uniaxial strain test machine (Shimadzu, Japan) operating at a fixed velocity strain rate of 5 mm min^{-1}. To measure the shear stress between the membranes and electrodes, Flat CCM and Patterned CCM laminates were prepared by thermally patterning a Nafion film with a polyethylene terephthalate (PET) backing onto the IrO_2 electrode-coated sides of the membranes to prevent electrode detachment during testing. Each laminate—whether flat or patterned—comprised a membrane/electrode/Nafion/PET film assembly with a test area of 2 cm × 2 cm, and a constant strain rate of 5 mm min^{-1} was applied. To evaluate water uptake and dimensional changes of the membrane, the membranes were cut into 1 cm × 1 cm segments and dried in a vacuum oven at 80 °C for 12 hours. After cooling to room temperature, each value of initial length, thickness, and mass was obtained. To compare the dimensional change and water uptake at different soaking conditions for membrane patterning, the membranes were soaked with distilled water at 30, 50, and 80°C for 4 h, and the final length, thickness, and mass were measured. The dimensional changes and water uptake were calculated by comparing their initial values.[2] To measure the ion conductivity of the prepared membranes, patterned and pristine membranes were cut into 1 cm × 4 cm sizes and placed in contact with four Pt wires spaced 1 cm apart. The assembly was tightly fastened and EIS measurements were conducted by supplying fully humidified nitrogen at 45°C. The resistance was extracted from the Nyquist plot, where the intersection point with the real axis at a high-frequency regime, and utilized to determine the in-plane conductivity of the membranes.[2]

$$\text{Ionic conductivity (mS cm}^{-1}\text{)} = \frac{L}{R \times A}$$

where L, R, and A represent the width, resistance, and thickness of the membrane, respectively.

Supplementary Note 1. Optimization of key parameters for the patterning process.

To address the impact of the two key parameters (patterning pressure and temperature) on the final interfacial structure of the patterned AEM, we conducted a series of experiments.

Patterning Pressure: We investigated the effect of patterning pressure by varying it between 1 MPa and 20 MPa. Pressures below 10 MPa (1 and 5 MPa) resulted in incomplete pattern formation, as the mesh structure did not fully transfer to the membrane (**Figure S5**). In contrast, at pressures of 10 MPa or higher, the pattern was clearly and consistently formed on the membrane surface, creating a reliable 3D interlocked interface. This indicates that, even though the Young's modulus and tensile strength of the plasticized hydrated AEM decreased from 981 MPa to 89.5 MPa and from 45.7 MPa to 7.11 MPa (**Figure 2b**), respectively, substantial mechanical pressure is still required to induce creep deformation in the AEM. Based on these findings, we selected 10 MPa as the optimal patterning pressure to balance effective patterning with minimal risk of mechanical stress to the membrane.

Patterning Temperature: We investigated the patterning temperature under the optimized pressure of 10 MPa and a patterning time of 2 hours, with temperatures varied at 30°C, 50°C, and 80°C. During the patterning process, permanent structural creep deformation was induced, preventing substantial immediate creep recovery upon mold removal by enhancing the AEM's inherent mechanical rigidity through a dehydration process (i.e., reducing the AEM's flexibility). At a patterning temperature of 80°C for 2 hours, high pattern fidelity was achieved, fully reflecting the original structural characteristics of the mesh mold. However, as the patterning temperature decreased to 50°C and 30°C, the pattern fidelity significantly declined (**Figure S6**). This is because, under the 50°C and 30°C conditions, the AEM remains partially hydrated during the 2-hour patterning process, allowing the partially hydrated patterned AEM to recover its shape upon mold removal (i.e., instant creep recovery), thereby reducing the depth and stability of the pattern. In contrast, 80°C provided the best results, as this temperature facilitated full dehydration, ensuring that the membrane permanently retained the imprinted structure. Although higher temperatures over 80°C could achieve more rapid dehydration, they might compromise membrane stability. Therefore, an optimal patterning temperature of approximately 80°C was selected, as it allowed for both high pattern fidelity and recovery of the membrane's mechanical properties **(Figure S2**).

In summary, the optimal conditions (24-hour hydration at 80°C, 10 MPa patterning pressure, and 80°C dehydration during patterning) were crucial for achieving a stable, high-fidelity 3D patterned interface. This optimized process effectively enhanced the ion transport and interfacial stability at the membrane-electrode interface under AEMWE operating conditions.

Figure S1. Dimensional changes and water uptake under different hydration periods at 50°C and 80°C.

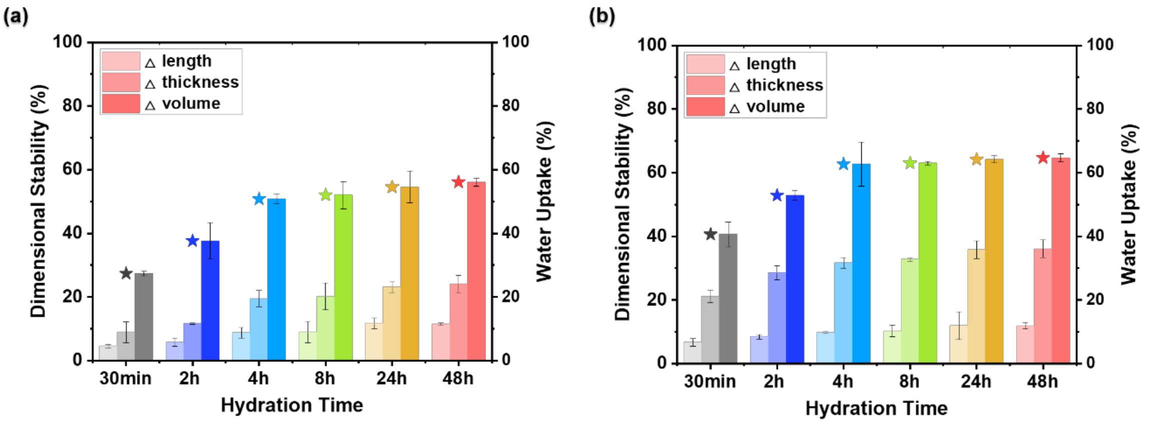

50℃	30min	2h	4h	8h	24h	48h
ΔL [f] (%)	4.532 ± 0.468	5.788 ± 1.212	8.773 ± 1.644	8.934 ± 3.311	11.6607 ± 1.7597	11.5528 ± 0.3283
Δt [g] (%)	8.892 ± 3.303	11.632 ± 0.273	19.411 ± 2.541	20.144 ± 4.246	23.1036 ± 1.675	24.084 ± 2.6557
ΔWU [e] (wt.%)	27.411 ± 0.745	37.658 ± 5.743	50.83 ± 1.551	52.026 ± 4.299	54.5623 ± 4.9615	56.107 ± 1.3396

80℃	30min	2h	4h	8h	24h	48h
ΔL [f] (%)	6.684 ± 1.159	8.416 ± 0.768	9.836 ± 0.473	10.275 ± 1.725	11.918 ± 4.408	11.917 ± 0.955
Δt [g] (%)	21.115 ± 1.962	28.51 ± 2.26	31.667 ± 1.667	32.778 ± 0.556	35.803 ± 2.833	36.09 ± 2.757
ΔWU [e] (wt.%)	40.632 ± 3.8975	52.849 ± 1.4987	62.72 ± 6.946	63.01 ± 0.5102	64.1835 ± 1.114	64.712 ± 1.2456

Figure S2. Stress-strain curves of pristine and patterned AEM.

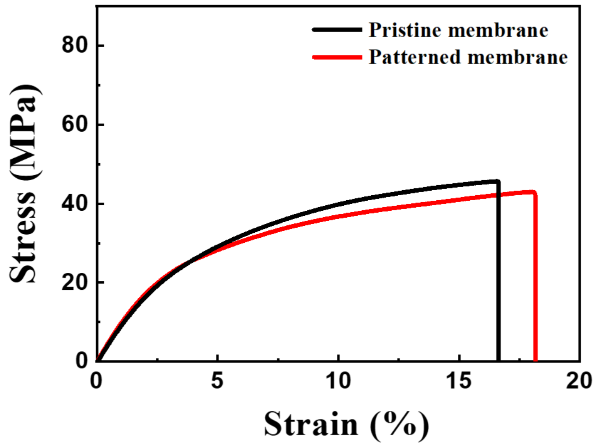

	Pristine membrane	Patterned membrane
**t (μm)**	40	38.74
**Tensile Strength (MPa)**	45.68	42.97
**Young's Modulus (MPa)**	980.6	1084.2
**Elongation to Break (%)**	16.632	18.166

Figure S3. Morphological characteristics of SUS316 mesh with (a) optical image and (b-c) 3D laser profile images of normal view (b) and titled view (c).

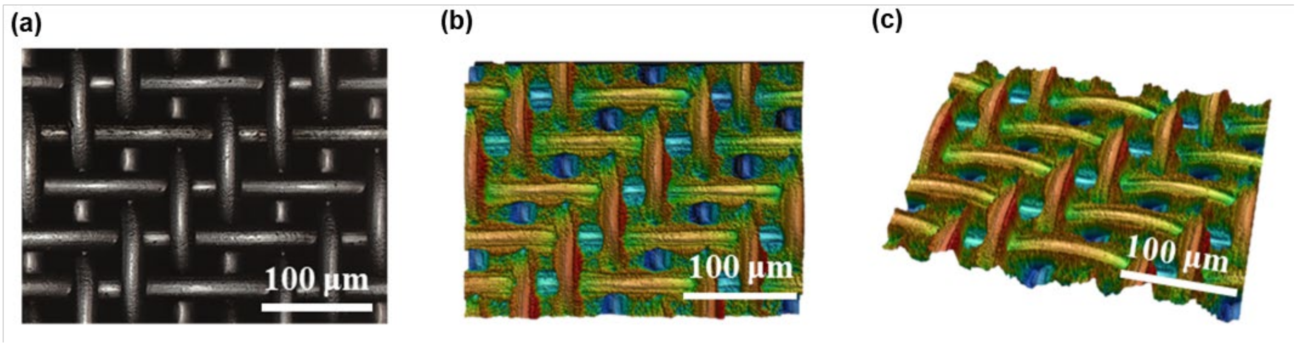

Figure S4. Digital camera images of SUS 316 mesh in roll-type configuration.

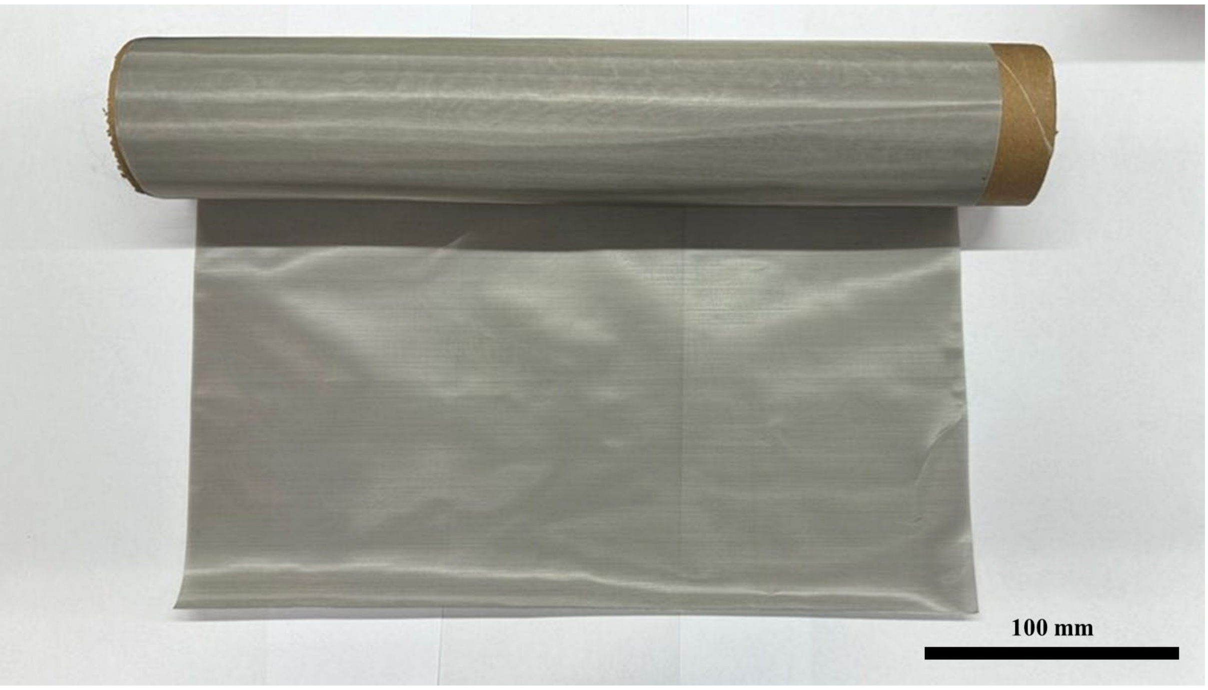

Figure S5. (a-d) Surface and (e-h) cross-sectional SEM images of patterned AEM under varying patterning pressures at 80°C for 2 hours. AEMs were hydrated at 80°C for 24 h before patterning.

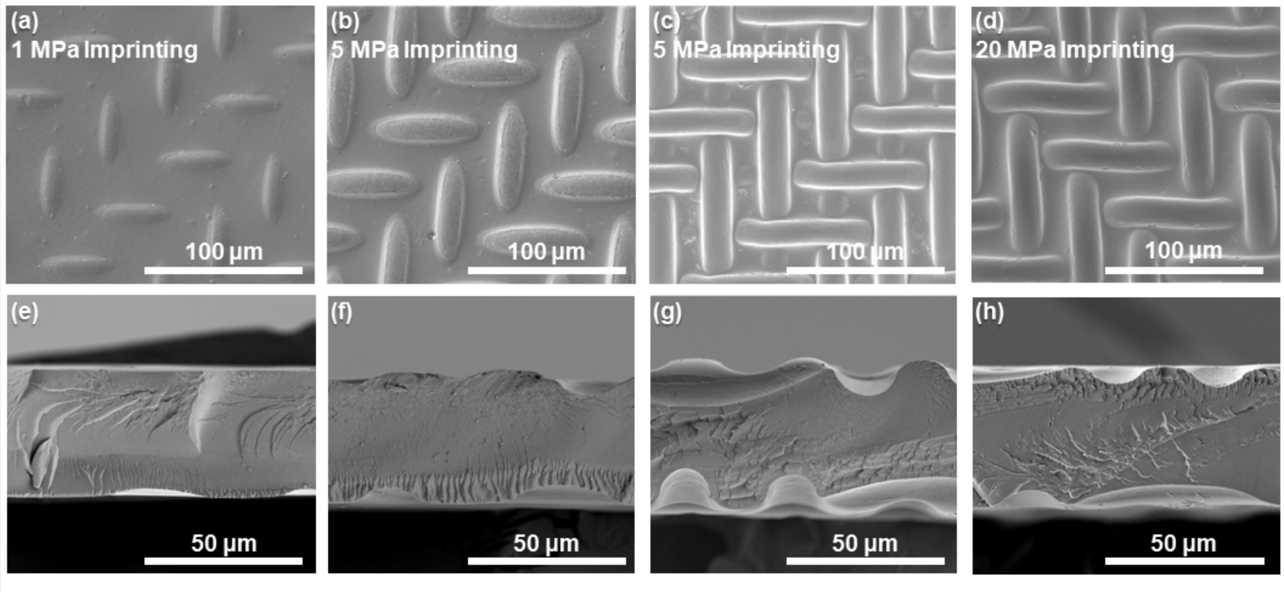

Figure S6. (a-d) Surface and (e-h) cross-sectional SEM images of patterned AEM under varying patterning temperatures at 10 MPa for 2 hours. AEMs were hydrated at 80℃ for 24 h before patterning.

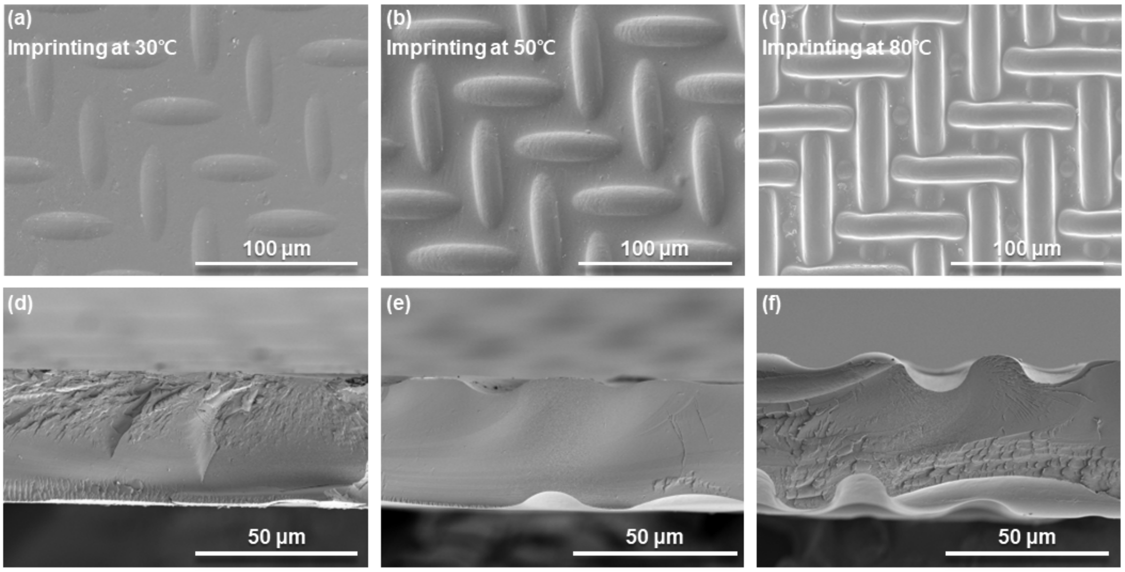

Figure S7. Optical and 3D laser profile images of patterned AEMs with different hydration conditions of (a) 30℃, (b) 50℃, and (c) 80℃.

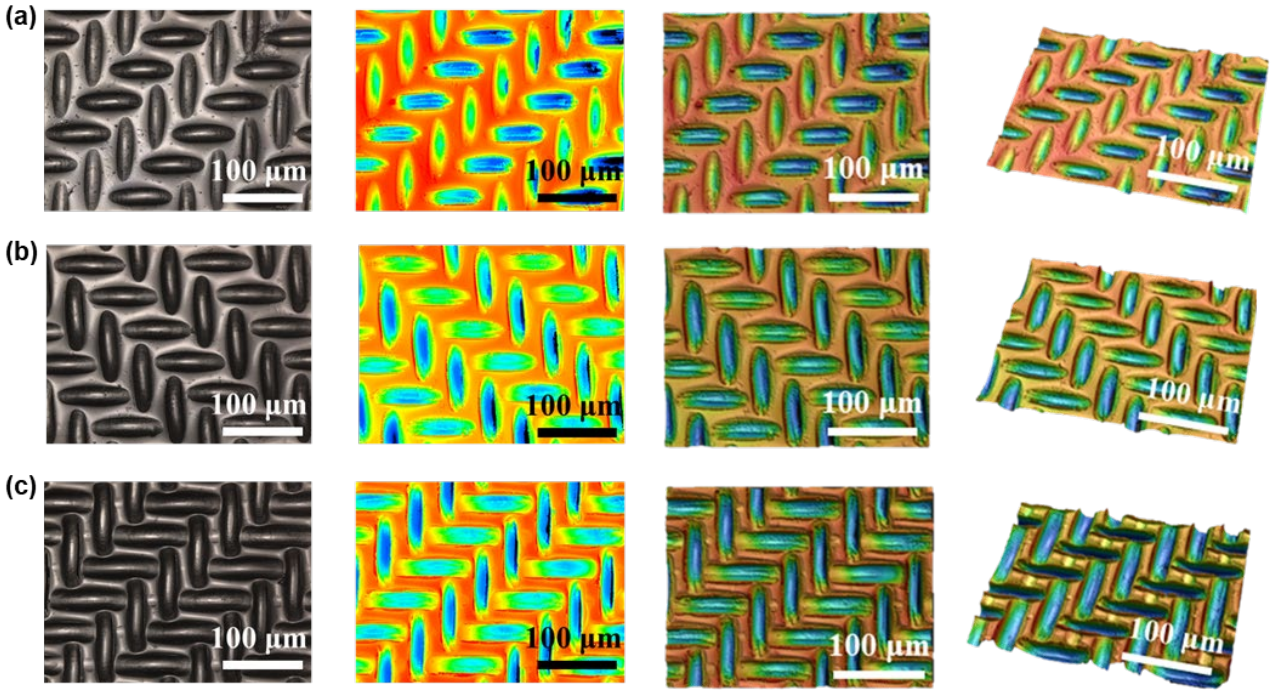

Figure S8. (a-c) Surface and cross-sectional SEM images of patterned AEMs with different hydration temperatures of (a) 30℃, (b) 50℃, and (c) 80℃.

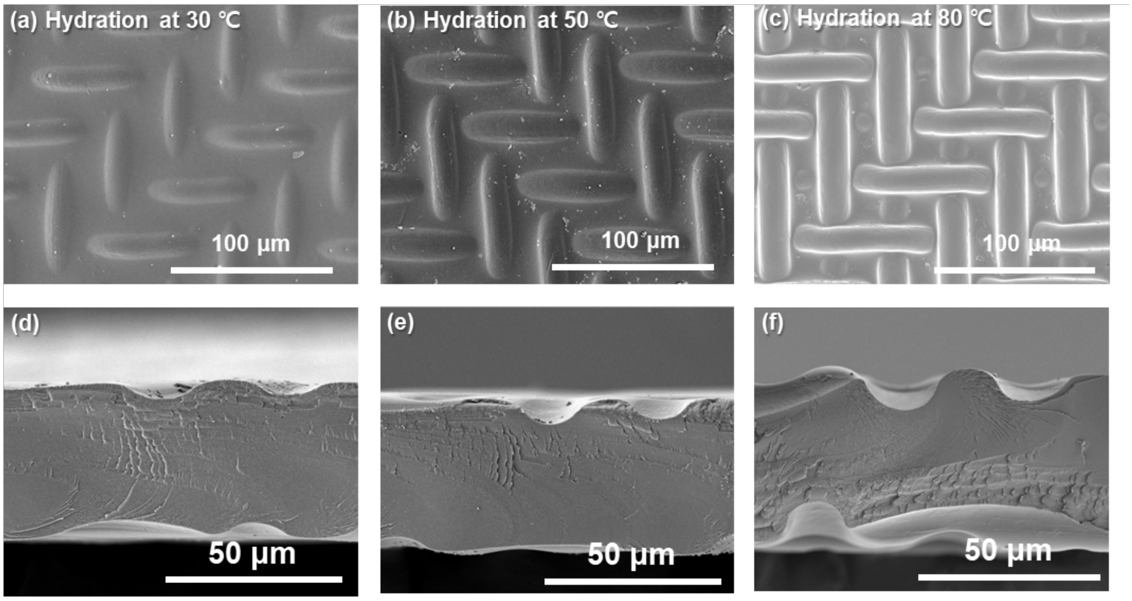

Figure S9. (a-c) Polarization curves of Flat CCM and Patterned CCM under pure water-fed conditions at different operating temperatures: (a) 45°C, (b) 60°C, and (c) 80°C. (d) Comparison of performance at 1.9 V.

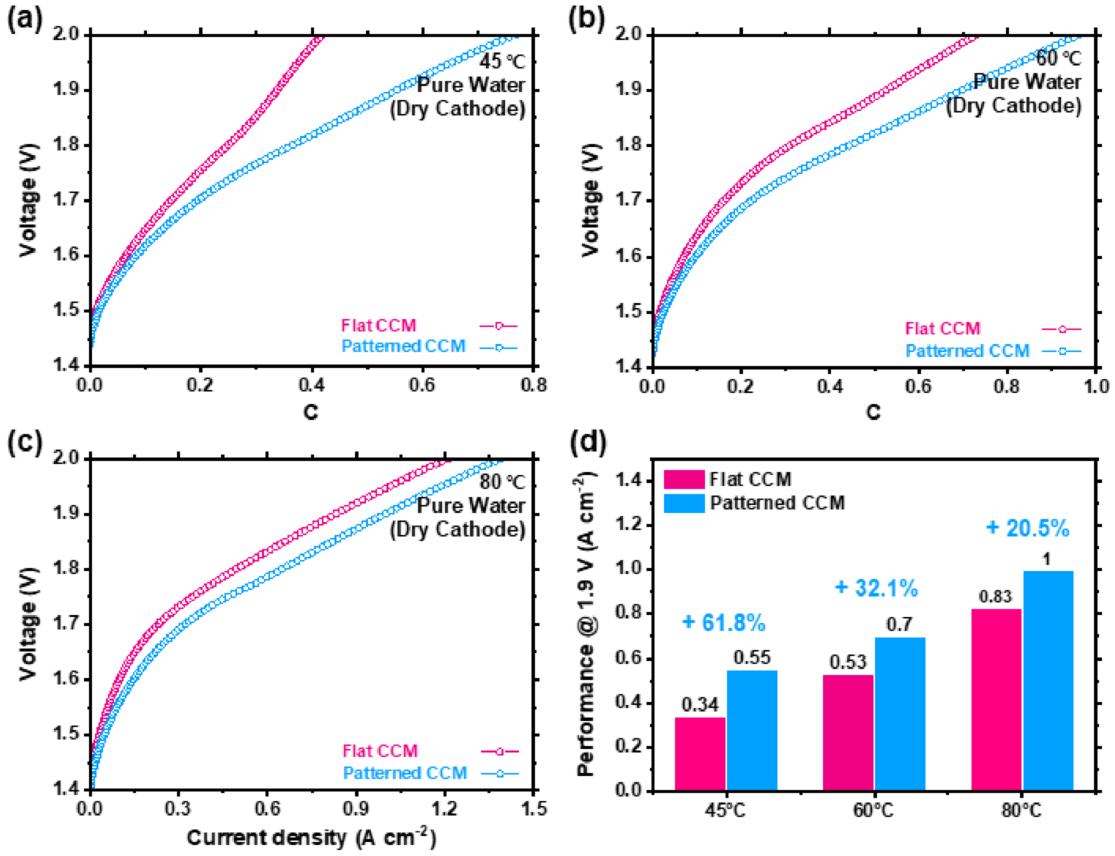

Figure S10. Polarization curves of MEAs with different ionomer to catalyst ratios in 1.0 M KOH, 0.1 M KOH, and pure water, respectively, at a cell temperature of 45°C.

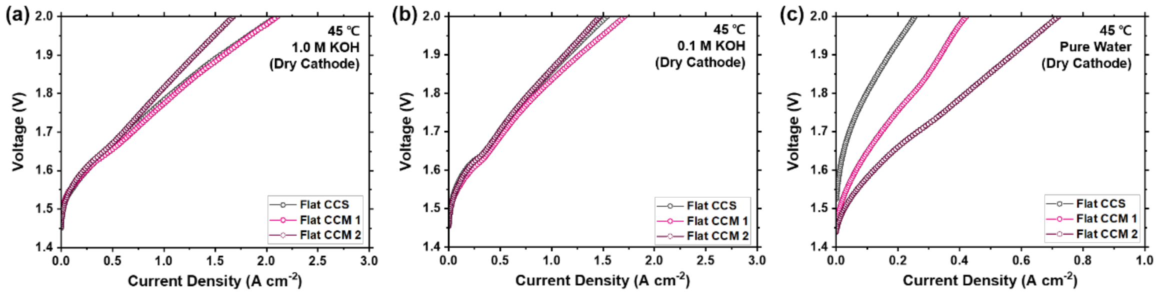

		IV performance (A cm^{-2}) (@1.9V)		
**Ionomer to Catalyst Ration (w/w)**	**MEA**	**1.0 M KOH**	**0.1 M KOH**	**DI**
**0.1**	**Flat CCM 1**	1.585	1.270	0.338
	**Patterned CCM**	1.886	1.629	0.563
**0.2**	**Flat CCM 2**	1.302	1.128	0.563

Figure S11. Polarization curves of patterned CCMs with different patterning temperatures in 1.0 M KOH, 0.1 M KOH, and pure water, respectively, at a cell temperature of 45°C.

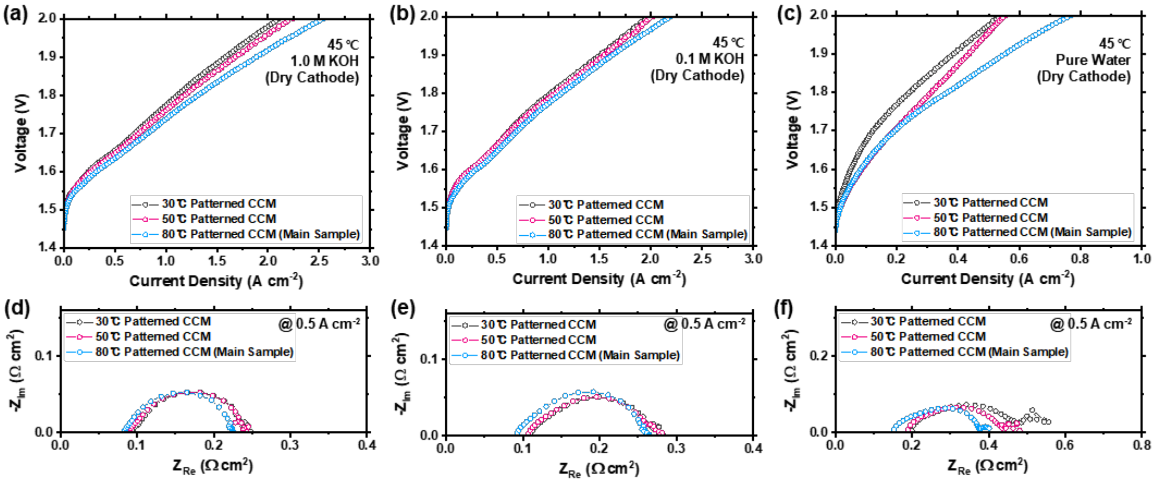

Figure S12. Cyclic voltammetry conducted at voltage from 0.4 to 0.6 V at scan rates of 20, 40, 60, 80 and 100 mV s^{-1} for the different MEAs with various electrolyte feed conditions.

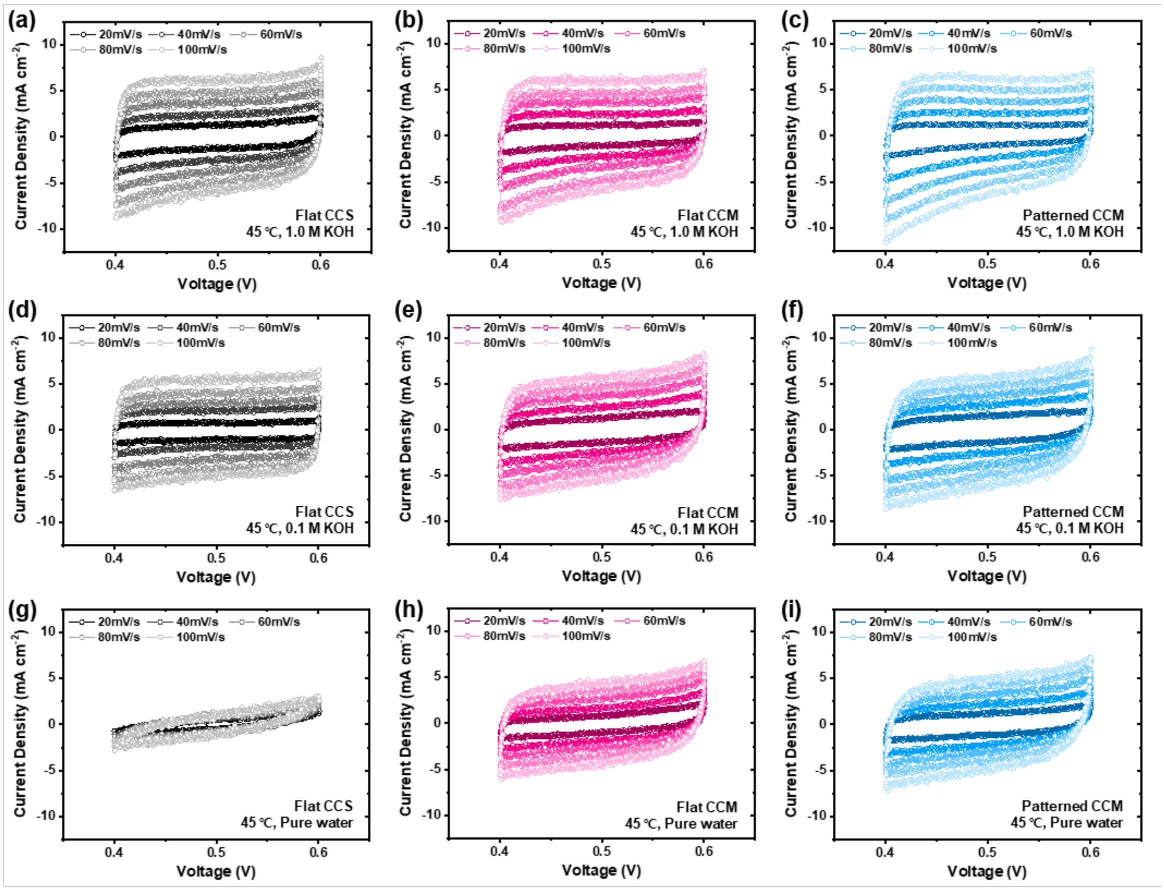

Figure S13. Cross-sectional SEM images of (a) Flat CCM and (b) Patterned CCM after durability test.

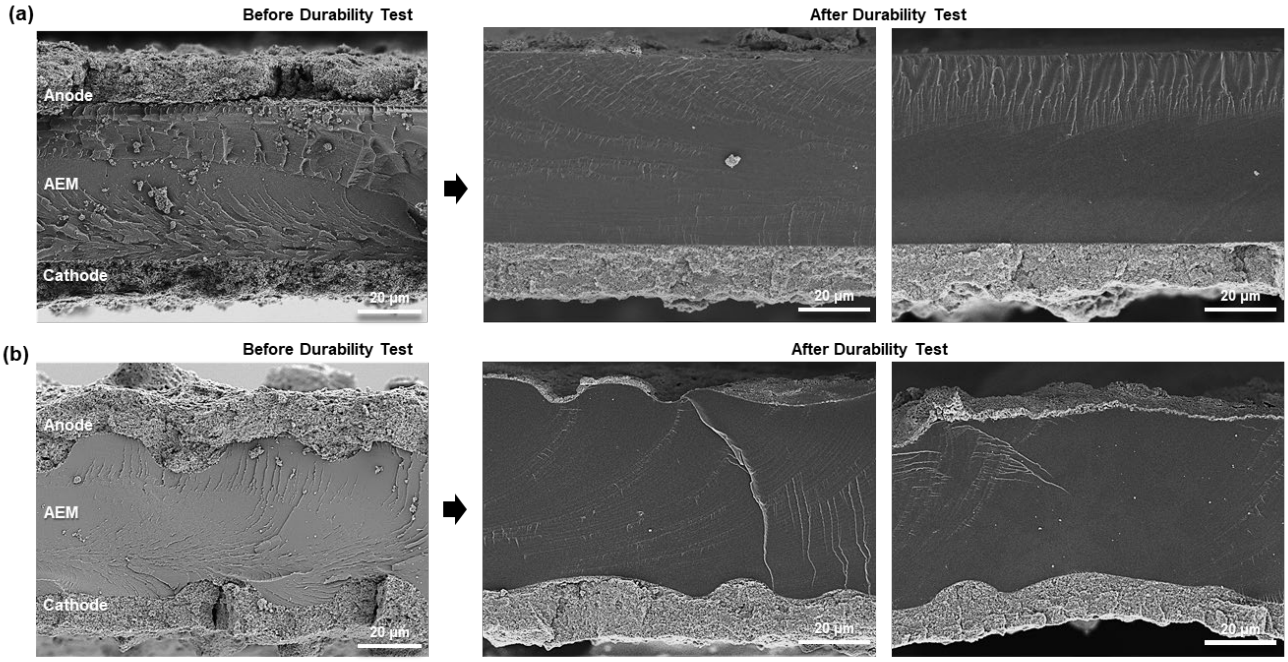

Figure S14. (a) Maximum shear stress of the prepared laminates with different membranes: pristine AEM and patterned AEM. (b) Digital camera images of the prepared laminates after the shear stress test, along with the corresponding surface and cross-sectional SEM images

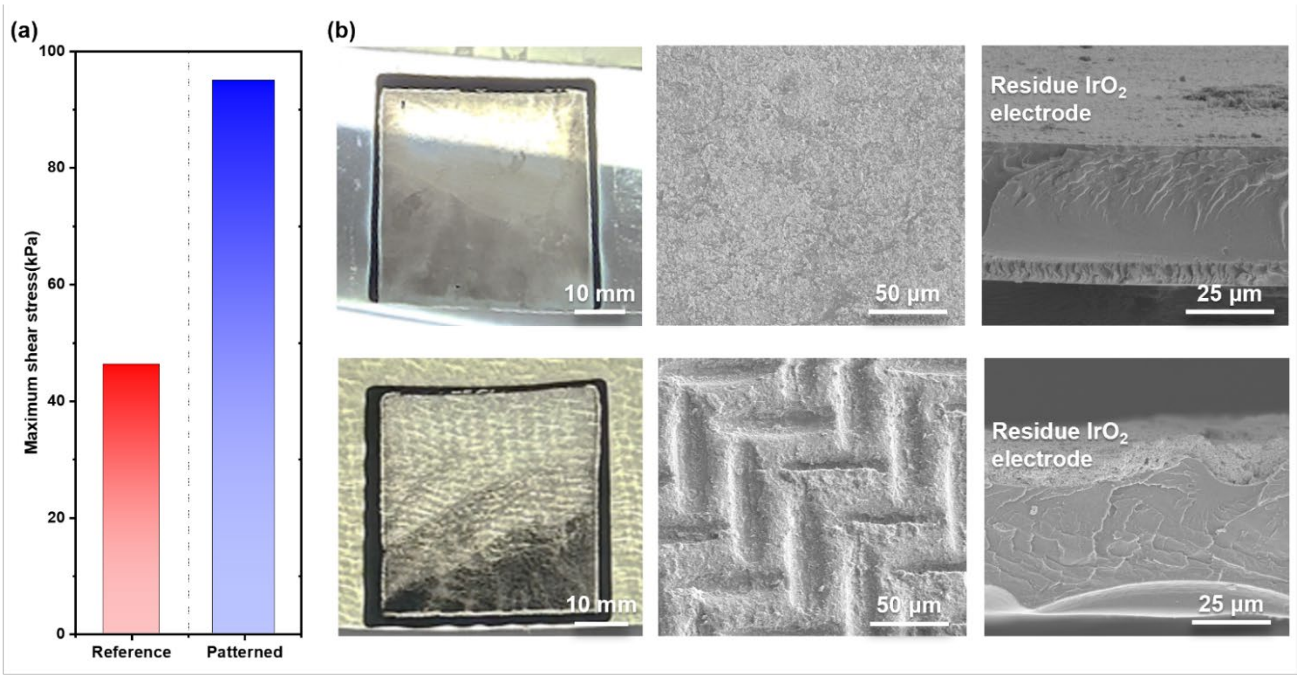

Figure S15. (a) Surface and (b) Cross-sectional SEM images of one-side patterned HQPC-TMA-2.4 AEM. (c) Cross-sectional SEM images of MEAs with one-side patterned HQPC-TMA-2.4 AEM.

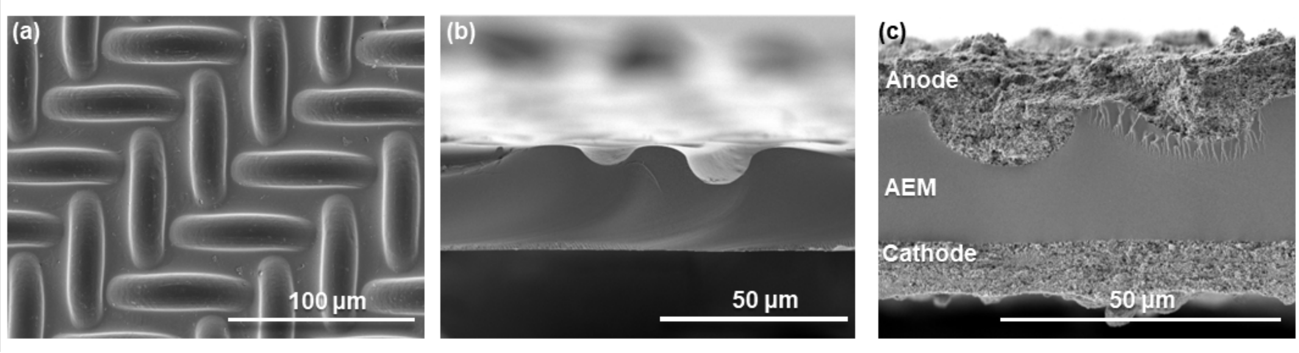

Figure S16. Nyquist and Bode plots of total cell impedance measured at 0.5, 1.0, and 2.5 A cm^{-2} of HQPC-TMA-2.4-based MEAs in (a-c) 1.0 M KOH-fed, and (d-j) pure water-fed condition at 60°C.

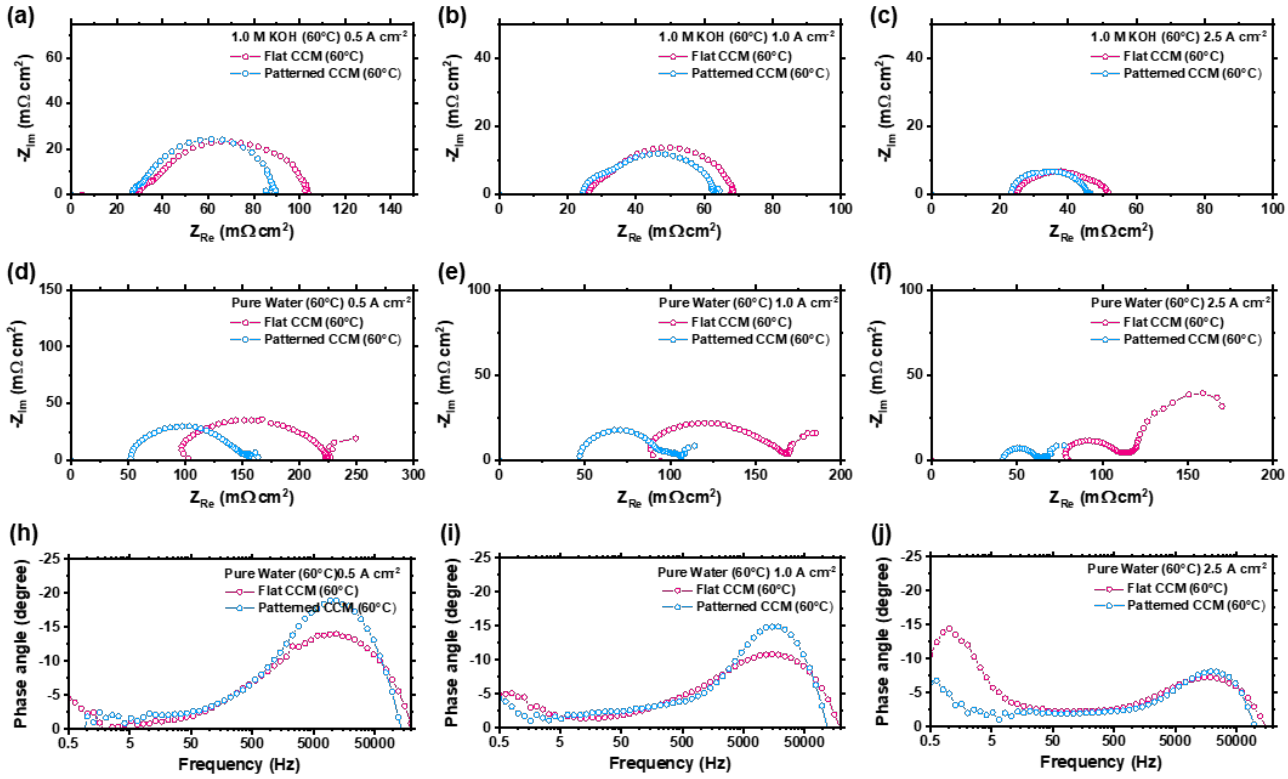

Figure S17. (a) Polarization curves of Flat CCM and Patterned CCM under pure water-fed conditions at different operating temperatures of 60°C and 80°C. (b) Comparison of performance at 2.0 V.

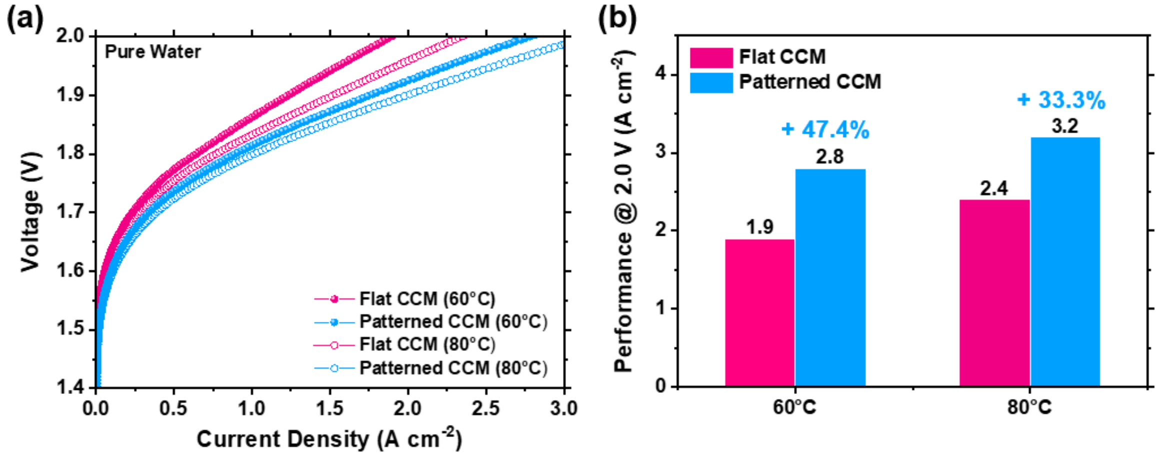

Figure S18. Comparison of the cell performance and durability of state of the art pure-water-fed AEMWE at 1.9 V reported in the literature.

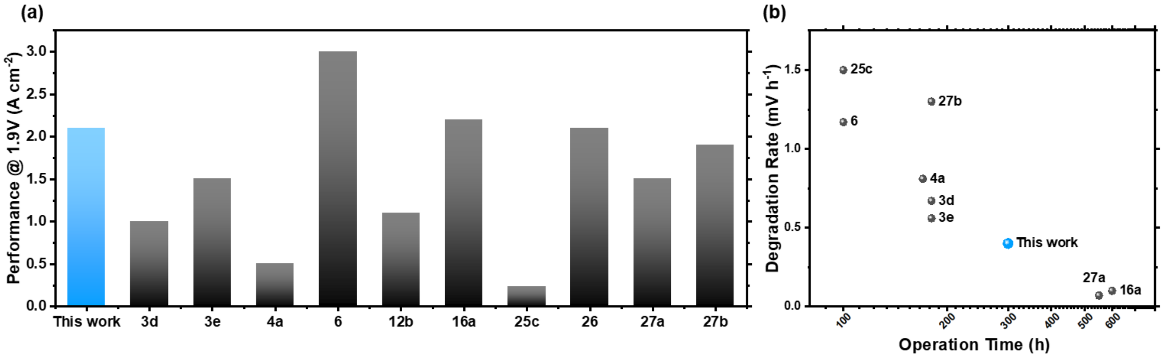

Figure S19. (a) Preparation of large-sized patterned HQPC-TMA-2.4 AEM for constructing CCM. (b-c) Surface (b) and cross-sectional (b) SEM images of patterned HQPC-TMA-2.4 AEM with 30 μm thickness.

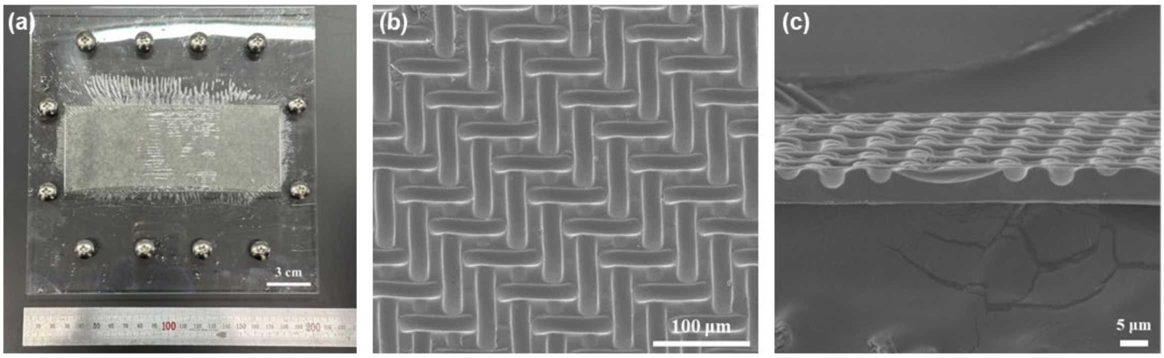

Figure S20. Area-specific polarization curves of small-size cell (active area of 5 cm^2) and large-size cell (active area of 68.75 cm^2) with patterned HQPC-TMA-2.4 membrane in 1.0 M KOH-fed and pure water-fed conditions.

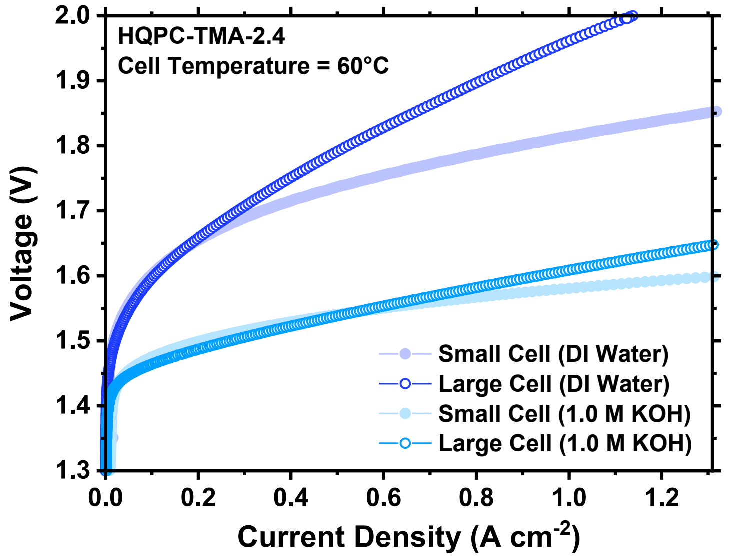

Table S1. Comparison of dimensional changes and water uptake characteristics of pristine AEM and Patterned AEM under hydration condition of 50°C and 80 °C.

**Temperature of 50℃**	**Ref**	**Pattern**
**ΔL (%)**	11.7 ± 1.8	11.1 ± 3.9
**Δt (%)**	23.1 ± 1.7	-
**ΔWU (wt.%)**	54.6 ± 5.0	53.6 ± 3.5
**Temperature of 80°C**	**Ref**	**Pattern**
**ΔL (%)**	11.9 ± 4.4	11.5 ± 0.1
**Δt (%)**	35.8 ± 2.8	-
**ΔWU (wt.%)**	64.2 ± 1.1	63.1 ± 1.4

Table S2. In-plane proton conductivity of pristine and patterned AEMs.

	**Pristine membrane**	**Patterned membrane**
R (Ω)	2250	2220
t (μm)	40	38.74
σ (mS cm^{-1})	111.11	116.28

Table S3. Comparison of the performance of pure water-fed AEMWEs using commercial AEMs.[3]

Ref	**AEM (Thickness, μm)**	**Anode catalyst [mg cm^{-2}]**	**Cathode catalyst [mg cm^{-2}]**	**Ionomer**	**Porous Transport Layer**	**T [°C]**	**Feeding condition**	**Performance at 1.9 V [A cm^{-2}]**
This Work	Mesh-patterned PiperION-A40 (40 μm)	IrO_2 (CCM) [2.0 mg cm^{-2}]	Pt/C (CCM) [0.4 mg cm^{-2}]	PiperION-A5	An: Ti felt Ca: carbon paper	80	Dry Cathode	≈ 1.00
[3a]	PAP-TP-85 (20 μm)	Ni Fe LDH/nickel foam (CCS)	Ru_NiCr LDH-r (CCM) 0.64mg cm^{-2}	PAP-TP-85	An: Nickel foam Ca: carbon paper	80	Dry Cathode	≈0.82
[3a]	PAP-TP-85 (20 μm)	Ni Fe LDH /nickel foam (CCS)	47wt% Pt/C (CCM) [0.94 mg cm^{-2}]	PAP-TP-85	An: Nickel foam Ca: carbon paper	80	Dry Cathode	≈0.7
[3b]	PAP-TP-85 (20 μm)	20 wt.% Ir/C (CCS) [4.8 mg$_{IrO2}$ cm^{-2}]	47wt% Pt/C (CCM) [0.94 mg cm^{-2}]	PAP-TP-85	An: Ni foam Ca: TGP-H-60 Toray carbon paper	80	Dry Cathode	≈0.2
[3b]	PAP-TP-85 (20 μm)	self-supported Fe_xNi_yOOH-20F catalyst (CCS) (20 wt. %, [4.8 mg cm^{-2}]	47wt% Pt/C (CCM) [0.94 mg cm^{-2}]	PAP-TP-85	An: Ni foam Ca: TGP-H-60 Toray carbon paper	80	Dry Cathode	≈0.8
[3c]	PAP-TP-85 (40 μm)	stainless-steel woven mesh (CCS) [2.2 -2.7 mg cm^{-2}]	Pt black (CCS) [2.2 -2.7 mg cm^{-2}]	PAP-TP-85	An: woven stainless-steel mesh Ca: carbon paper	57	Dry Cathode	≈0.5
[3d]	PiperION-A40 (40 μm)	LSC-PP-OER49B (CCS) [3.5 mg cm^{-2}]	60wt% PtRu/C (CCS) [1.0 mg cm^{-2}]	PiperION-A5 Nafion	An: Ti PTL Ca: carbon paper PTL	80	Dry Cathode	≈0.855

[3e]	PiperION-A40 (40 µm)	Ir, Co e-beam deposition on stainless steel PTL (CCS) [1 mg cm^{-2}]	Pt (CCS) [2-3 mg cm^{-2}]	PAP-TP-85	An: stainless steel PTL Ca: carbon paper	56	Dry Cathode	0.125
[3f]	PAP-TP-85 (40 µm)	Co_3O_4 (CCS)	Pt-black (CCS)	PAP-TP-85	An: Ni PTE Ca: Toray carbon paper	55	Both feeding	≈0.3
[3g]	PAP-TP-85 (50 µm)	IrO_2 (CCS) [2.3-2.8 mg cm^{-2}]	Pt black (CCS) [2.3-2.8 mg cm^{-2}]	PAP-TP-85	An: stainless-steel mesh filter material Ca: Toray 090 carbon paper	55	Both feeding	≈0.6
[3g]	Sustanion (50 µm)	IrO_2 (CCS) [2.3-2.8 mg cm^{-2}]	Pt black (CCS) [2.3-2.8 mg cm^{-2}]	Sustainion	An: stainless-steel mesh filter material Ca: Toray 090 carbon paper	55	Both feeding	≈0.38
[3h]	FAA-3-50 (50 µm)	IrO_2 (CCM) [2.0 mg cm^{-2}]	40wt% PtC (CCM) [0.4 mg cm^{-2}]	FAA-3	An: JNTG-30-A6H GDL Ca: JNTG-30-A6H GDL	70	-	≈0.65
[3i]	FAA-3-50 (50 µm)	IrO_2 (CCM) [2.0 mg cm^{-2}]	40wt% PtC (CCM) [0.4 mg cm^{-2}]	FAA-3	An: Titanium felt Ca: carbon paper with microporous layer	70	Both feeding	≈0.11
[3j]	FAA-3-130 (50 µm)	IrO_2 (CCS) [3.0 mg cm^{-2}]	Pt Black (CCS) [3.0 mg cm^{-2}]	FAA-3	An: Pt coated sintered titanium felt Ca: carbon paper	50	-	≈0.15

	with microporous layer

Reference

[1] S. Kim, S. H. Yang, S. H. Shin, H. J. Cho, J. K. Jang, T. H. Kim, S. G. Oh, T. H. Kim, H. Han, J. Y. Lee, *Energ Environ Sci* **2024**. https://doi.org/10.1039/d4ee01003e.

[2] E. Choi, H. J. Kang, Y. Jang, J. H. Kim, H. S. Ahn, S. M. Kim, S. Jang, *Adv Mater Technol-Us* **2024**, *9*. https://doi.org/10.1002/admt.202301191.

[3] a)J. H. Yang, S. X. Yang, L. L. An, J. Zhu, J. W. Xiao, X. Zhao, D. L. Wang, *Acs Catal* **2024**, *14*, 3466-3474. https://doi.org/10.1021/acscatal.3c05550; b)J. W. Xiao, A. M. Oliveira, L. Wang, Y. Zhao, T. Wang, J. H. Wang, B. P. Setzler, Y. S. Yan, *Acs Catal* **2021**, *11*, 264-270. https://doi.org/10.1021/acscatal.0c04200; c)R. A. Krivina, G. A. Lindquist, S. R. Beaudoin, T. N. Stovall, W. L. Thompson, L. P. Twight, D. Marsh, J. Grzyb, K. Fabrizio, J. E. Hutchison, S. W. Boettcher, *Adv Mater* **2022**, *34*. https://doi.org/10.1002/adma.202203033; d)L. Osmieri, Y. H. He, H. T. Chung, G. McCool, B. Zulevi, D. A. Cullen, P. Zelenay, *J Power Sources* **2023**, *556*. https://doi.org/10.1016/j.jpowsour.2022.232484; e)M. Kwak, K. Ojha, M. K. Shen, S. W. Boettcher, *Acs Energy Lett* **2024**, *9*, 1025-1034. https://doi.org/10.1021/acsenergylett.3c02620; f)G. A. Lindquist, J. C. Gaitor, W. L. Thompson, V. Brogden, K. J. T. Noonan, S. W. Boettcher, *Energ Environ Sci* **2023**, *16*, 4373-4387. https://doi.org/10.1039/d3ee01293j; g)G. A. Lindquist, S. Z. Oener, R. Krivina, A. R. Motz, A. Keane, C. Capuano, K. E. Ayers, S. W. Boettcher, *Acs Appl Mater Inter* **2021**, *13*, 51917-51924. https://doi.org/10.1021/acsami.1c06053; h)M. S. Cha, J. E. Park, S. Kim, S. H. Han, S. H. Shin, S. H. Yang, T. H. Kim, D. M. Yu, S. Y. So, Y. T. Hong, S. J. Yoon, S. G. Oh, S. Y. Kang, O. H. Kim, H. S. Park, B. Bae, Y. E. Sung, Y. H. Cho, J. Y. Lee, *Energ Environ Sci* **2020**, *13*, 3633-3645. https://doi.org/10.1039/d0ee01842b; i)S. Y. Kang, J. E. Park, G. Y. Jang, O. H. Kim, O. J. Kwon, Y. H. Cho, Y. E. Sung, *Int J Hydrogen Energ* **2022**, *47*, 9115-9126. https://doi.org/10.1016/j.ijhydene.2022.01.002; j)D. Y. Xu, M. B. Stevens, M. R. Cosby, S. Z. Oener, A. M. Smith, L. J. Enman, K. E. Ayers, C. B. Capuano, J. N. Renner, N. Danilovic, Y. G. Li, H. Z. Wang, Q. H. Zhang, S. W. Boettcher, *Acs Catal* **2019**, *9*, 7-15. https://doi.org/10.1021/acscatal.8b04001.
